# High-Throughput Analysis of Gene Essentiality and Sporulation in Clostridium difficile

**DOI:** 10.1128/mBio.02383-14

**Published:** 2015-02-24

**Authors:** Marcin Dembek, Lars Barquist, Christine J. Boinett, Amy K. Cain, Matthew Mayho, Trevor D. Lawley, Neil F. Fairweather, Robert P. Fagan

**Affiliations:** ^a^ MRC Centre for Molecular Bacteriology and Infection, Imperial College London, London, United Kingdom; ^b^ Institute for Cell and Molecular Biosciences, Newcastle University, Newcastle upon Tyne, United Kingdom; ^c^ Institute for Molecular Infection Biology, University of Würzburg, Würzburg, Germany; ^d^ Wellcome Trust Sanger Institute, Hinxton, United Kingdom; ^e^ Department of Molecular Biology and Biotechnology, University of Sheffield, Sheffield, United Kingdom; Harvard Medical School

## Abstract

Clostridium difficile is the most common cause of antibiotic-associated intestinal infections and a significant cause of morbidity and mortality. Infection with C. difficile requires disruption of the intestinal microbiota, most commonly by antibiotic usage. Therapeutic intervention largely relies on a small number of broad-spectrum antibiotics, which further exacerbate intestinal dysbiosis and leave the patient acutely sensitive to reinfection. Development of novel targeted therapeutic interventions will require a detailed knowledge of essential cellular processes, which represent attractive targets, and species-specific processes, such as bacterial sporulation. Our knowledge of the genetic basis of C. difficile infection has been hampered by a lack of genetic tools, although recent developments have made some headway in addressing this limitation. Here we describe the development of a method for rapidly generating large numbers of transposon mutants in clinically important strains of C. difficile. We validated our transposon mutagenesis approach in a model strain of C. difficile and then generated a comprehensive transposon library in the highly virulent epidemic strain R20291 (027/BI/NAP1) containing more than 70,000 unique mutants. Using transposon-directed insertion site sequencing (TraDIS), we have identified a core set of 404 essential genes, required for growth *in vitro*. We then applied this technique to the process of sporulation, an absolute requirement for C. difficile transmission and pathogenesis, identifying 798 genes that are likely to impact spore production. The data generated in this study will form a valuable resource for the community and inform future research on this important human pathogen.

## IMPORTANCE

Clostridium difficile is a common cause of potentially fatal intestinal infections in hospital patients, particularly those who have been treated with antibiotics. Our knowledge of this bacterium has been hampered by a lack of tools for dissecting the organism. We have developed a method to study the function of every gene in the bacterium simultaneously. Using this tool, we have identified a set of 404 genes that are required for growth of the bacteria in the laboratory. C. difficile also produces a highly resistant spore that can survive in the environment for a long time and is a requirement for transmission of the bacteria between patients. We have applied our genetic tool to identify all of the genes required for production of a spore. All of these genes represent attractive targets for new drugs to treat infection.

## INTRODUCTION

Clostridium difficile is a Gram-positive, spore-forming anaerobe and the leading cause of nosocomial, antibiotic-associated diarrhea (1). Infection typically occurs among patients whose intestinal microbiota has been disrupted by prolonged treatment with broad-spectrum antibiotics, allowing the pathogen to colonize the compromised gastrointestinal tract (2). C. difficile infection (CDI) is most common in the nosocomial setting, but the incidence of community-associated infections is increasing (3). Two large cytotoxins are responsible for the clinical manifestations of CDI, which range from mild, self-limiting diarrhea to severe, often fatal inflammatory complications, such as pseudomembranous colitis or toxic megacolon (4).

The spore is the primary infectious agent, and recent studies have shown that a mutant strain of C. difficile unable to produce Spo0A, the master regulator of sporulation, is unable to efficiently persist in the environment and transmit disease (5). Due to their multilayered structure, spores are extremely robust and resistant to both chemical and physical insults, thus providing the mechanism by which C. difficile evades the potentially fatal consequences of exposure to heat, oxygen, alcohol, and certain disinfectants (6). Spores shed in feces are therefore difficult to eradicate and can persist in health care facilities for extended periods of time leading to infection or reinfection of individuals through inadvertent ingestion of contaminated material (7).

Sporulation is an ancient bacterial cell differentiation program that is largely conserved among *Clostridiales* and *Bacillales*, particularly with regard to the key regulatory components Spo0A and the four sporulation-specific sigma factors, σ^E^, σ^F^, σ^G^, and σ^K^ (reviewed in reference 8). However, recent studies have highlighted several notable differences in the sporulation programs of Bacillus and Clostridium species. While both Spo0A and σ^H^ are present and required for sporulation in C. difficile (9), phosphorylation of Spo0A in C. difficile involves a simple two-component system unlike the complex phosphorelay that modulates Spo0A activity in Bacillus subtilis (8, 10). In C. difficile, the main periods of activity of the 4 cell type-specific sigma factors are similar but not identical to those in the B. subtilis model, with σ^F^ and σ^E^ controlling early stages of development and σ^G^ and σ^K^ governing late developmental events. However, the temporal segregation between the activities of the early and late-stage sigma factors is less stringent, and the cross talk between the forespore and the mother cell appears to be weaker, as the activity of σ^E^ is partially independent of σ^F^, and activation of σ^G^ and σ^K^ does not require σ^E^ and σ^G^, respectively (11–13). The C. difficile σ^E^, σ^F^, σ^G^, and σ^K^ regulons have now been identified (12, 13), each containing key representatives of the homologous pathways in B. subtilis (14). This core set of 228 sporulation genes corresponds to about half the number of genes under the control of cell type-specific sigma factors in B. subtilis (15–17). This may be a reflection of the more ancestral mechanism of sporulation proposed for clostridia (8, 18).

Understanding the genetic and molecular bases of C. difficile pathogenicity is a crucial step in the development of effective therapeutics. A number of methods for directed gene inactivation are now available (19–22), allowing for reverse genetic studies, in which the exact role of a gene, hypothesized to be important in a particular phenotype, can be elucidated experimentally. However, reverse genetic studies are limited in that they are always based on premade assumptions regarding the function of a particular gene. An alternative approach involves using random mutant libraries aimed at identifying the genetic basis of a particular phenotype without making any assumptions about the genes involved (23). This is typically achieved through transposon-mediated mutagenesis, creating random mutant pools that can then be screened to identify genes involved in a particular phenotype across the whole genome. Random transposon mutagenesis can then be combined with high-throughput sequencing methods to allow simultaneous screening of saturating transposon libraries without the need to isolate individual mutants (reviewed in references 24 and 25). The *mariner* transposable element inserts randomly into TA target sites via a cut-and-paste mechanism (26), making it particularly useful in low-GC organisms such as C. difficile. A *mariner*-based transposon delivery system has been developed for use in C. difficile (27). However, the relative inefficiency of plasmid delivery into C. difficile by conjugation and a lack of effective control of the timing of transposition limited the practical size of the transposon library that could be generated.

Here we describe the construction of the first comprehensive high-density transposon mutant library in C. difficile using a novel conditional *mariner* delivery vector. We have applied transposon-directed insertion site sequencing (TraDIS [28]) to identify the core C. difficile essential genome required for *in vitro* growth and have also identified a large number of genes required for sporulation.

## RESULTS

### Construction of an efficient transposon mutagenesis system for C. difficile.

Early versions of a tetracycline-inducible expression system for C. difficile (29) displayed plasmid instability upon addition of anhydrotetracycline, a nonantibiotic analogue of tetracycline. In this study, we have exploited this serendipitous plasmid instability to generate a conditional C. difficile plasmid for the delivery of a *mariner* transposon. The tetracycline-inducible system consists of a pair of divergent promoters, each with an overlapping *tet* operator sequence (TetO). One of the promoters, P_tetR_, drives expression of *tetR*, encoding a transcriptional regulator that binds to the *tet* operators to repress both promoters. The second promoter, P_tet_, is available to drive the expression of any gene of interest. Tetracycline (or anhydrotetracycline) induces a conformational change in TetR that prevents binding to the operator and relieves repression (30). Under normal conditions, TetR is expressed at low levels, maintaining repression of the system, and induction with tetracycline greatly increases transcription from both promoters. In plasmid pRPF177, the P_tetR_ promoter is oriented toward the C. difficile pCD6 origin of replication (see Fig. S1A in the supplemental material [31]), and this plasmid displays induction-dependent instability. Addition of a transcriptional terminator sequence between the *tetR* gene and the CD6 origin of replication (pRPF185 [29]) prevents this tetracycline-dependent instability, demonstrating that transcriptional read-through from P_tetR_ into the origin of replication causes the instability.

The conditional plasmid pRPF177 was used as the basis for the construction of a *mariner* delivery system. A codon-optimized *Himar1* transposase gene was cloned downstream of the P_tet_ promoter, and a custom transposon was assembled by adding the *mariner* inverted terminal repeat sequences (32) either side of an *ermB* erythromycin resistance gene. The resulting *mariner* delivery plasmid, pRPF215, retains tetracycline-dependent conditional replication while allowing tightly regulated transposition of the *ermB* transposon (see Fig. S1B in the supplemental material).

To confirm tetracycline-dependent conditionality, a single culture of C. difficile 630Δ*erm* carrying pRPF215 was divided in two and then grown in TY broth (48) with and without anhydrotetracycline. After 13 generations, bacteria were spread on BHI agar with or without thiamphenicol to determine the proportion of cells retaining the plasmid. In the culture without anhydrotetracycline, more than 40% of bacteria were thiamphenicol resistant after 13 generations, whereas in the anhydrotetracycline-induced culture, no thiamphenicol-resistant bacteria were detectable (limit of detection, 10 CFU/ml; data not shown).

In order to determine the frequency of transposition, C. difficile strain 630Δ*erm* carrying pRPF215 was grown to mid-logarithmic phase in TY broth, and dilutions were spread on BHI agar plates containing either thiamphenicol to select for the plasmid or erythromycin and anhydrotetracycline to select for transposon mutants. Erythromycin-resistant mutants arose with a frequency of 1.18 × 10^−4^. It was also observed that induction of transposition resulted in the production of a wide range of colony morphologies, suggesting successful random transfer of the transposon (see Fig. S1C and S1D in the supplemental material). The frequency of transposition was higher in strain R20291 (1.5 × 10^−3^) than in strain 630Δ*erm*. To determine the frequency of spontaneous erythromycin resistance or mutation of the conditional plasmid replicon, an analogous experiment was carried out with C. difficile strain 630Δ*erm* carrying plasmid pRPF222, encoding a nonfunctional *Himar1* transposase. Erythromycin-resistant colonies arose with a frequency of 5.9 × 10^−8^; therefore, the frequency of spontaneous Erm^r^ mutants in a transposon library would be expected to be of the order of 4 × 10^−4^.

### Generation of a large C. difficile transposon mutant library.

**FIG 1:**
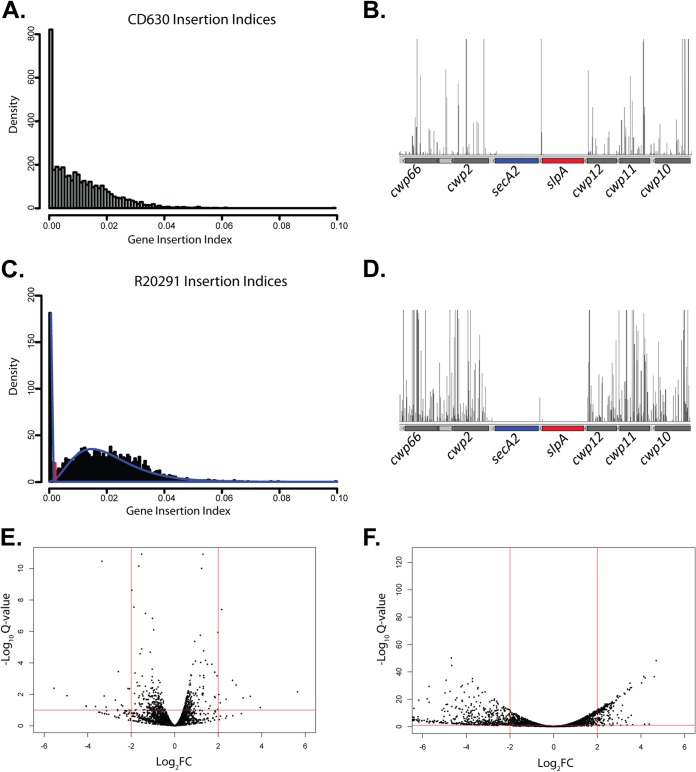
Identification of sporulation-specific genes in C. difficile. (A and C) Insertion index distribution plots from the C. difficile 630Δ*erm* (A) and R20291 (C) transposon libraries. The clear bimodal distribution in the R20291 plot (fitted gamma distributions in blue) allows differentiation of genes that do not tolerate transposon insertions in the sharp left-hand peak and genes containing insertions in the broad right-hand peak. (B and D) S-layer biogenesis locus. Each vertical line indicates the location of a unique transposon insertion, with height indicating the total number of Illumina reads at each insertion point. The *secA2* and *slpA* genes show no transposon insertions, which suggests that they are required for growth *in vitro*. (E and F) Changes in prevalence of the mutants from the initial transposon library after overnight growth (E) or sporulation (F). Red lines show the cutoff criteria of 10% false discovery rate (horizontal) and a log_2_ fold change (Log_2_FC) of 2 (vertical).

In order to test the suitability of pRPF215 to generate large transposon libraries in C. difficile, a proof-of-principle library was constructed in model strain 630Δ*erm*. Individual mutants were generated on solid media, and approximately 85,000 erythromycin-resistant colonies were pooled. Following genomic DNA (gDNA) isolation from the pooled library, transposon insertion sites were identified using TraDIS following the method developed by Langridge et al. (28). A total of 44,102 unique insertion sites were identified, an average of one insertion every 97 bp. Of the 3,897 annotated coding DNA sequences (CDSs), 736 showed no transposon insertions; however, the insertion density attained did not appear sufficient to saturate nonessential genes (Fig. 1A and B). Despite not reaching saturation, this proof-of-principle experiment clearly demonstrated the ease with which large transposon libraries can be created in C. difficile. To identify genes involved in sporulation and germination among the 3,161 CDSs containing transposon insertions, the library was sporulated on solid media, and the spores were purified on a HistoDenz gradient (see Fig. S2A in the supplemental material) and germinated in broth containing the bile acid germinant taurocholate. gDNA was extracted from the spore, and germinated libraries and transposon insertion sites were identified. Mutants incapable of sporulation would be absent from the purified spore population. Likewise, mutants that were capable of producing spores but incapable of germination would be absent from the germination library (Fig. S2B).

### Analysis of individual mutants.

In order to validate the identification of genes required for sporulation and germination, genes with fewer observed transposon insertions following sporulation or germination were identified in the 630Δ*erm* library by manual inspection of insertion plots using Artemis (33). Candidate genes with clear differences in insertion frequency were further analyzed using BLAST. Three candidate genes (CD630_01250, CD630_34940, and CD630_35670) that appeared to play a role in sporulation and one candidate gene (CD630_01060) that appeared to play a role in germination were chosen for further analysis. Each of these genes was annotated as or displayed homology to genes implicated in sporulation or germination in other species.

CD630_01060 encodes a putative *N*-acetylmuramoyl-l-alanine amidase that shares 37% amino acid sequence identity with CwlD from Bacillus subtilis strain 168. CwlD has been shown to be essential for muramic-δ-lactam formation during sporulation in Bacillus (34). CD630_01250 encodes a putative endopeptidase that shares 21.4% amino acid sequence identity with B. subtilis SpoIIQ, a σ^F^-dependent membrane protein that localizes to the forespore membrane and is essential for forespore integrity and late forespore gene expression in B. subtilis (35). CD630_34940 encodes a putative spore coat-associated protein sharing 32.6% amino acid sequence identity with B. subtilis YabP, disruption of which causes a late spore morphogenesis defect (36). Finally, CD630_35670 (*sipL*) encodes a 58.7-kDa protein, identified as a functional homologue of SpoVID, a key morphogenetic protein that directly interacts with SpoIVA and is required for the correct attachment of the spore coat to the forespore (37).

**FIG 2:**
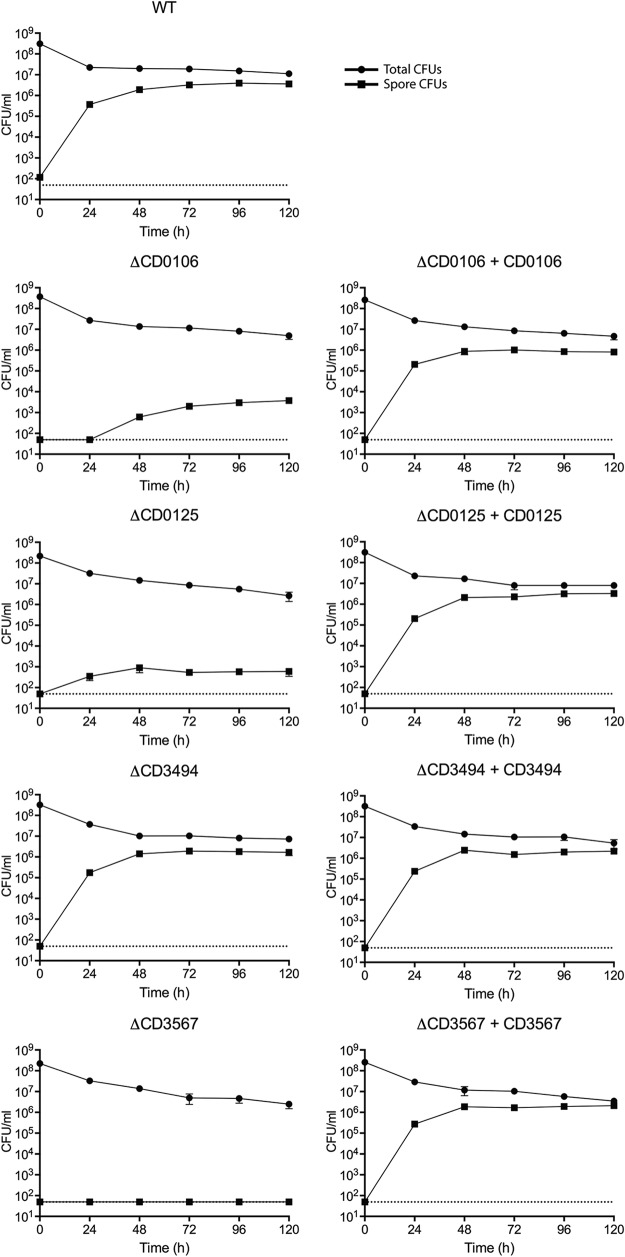
Sporulation efficiency of individual isogenic mutants. CD0106, CD0125, CD3494, and CD3567 deletion mutants were compared to strains complemented on the chromosome at the *pyrE* locus and the parental 630Δ*erm* strain (wild type [WT]). Stationary-phase cultures in BHIS broth were incubated anaerobically for 5 days, and samples were taken at 24-h intervals for analysis. Total cell numbers were determined by counting the number of CFU on BHIS agar containing the germinant taurocholate. Spore numbers were determined by the same method following incubation at 70°C for 30 min. Experiments were carried out in triplicate on biological duplicate samples. The means ± standard deviations (error bars) are shown.

**FIG 3:**
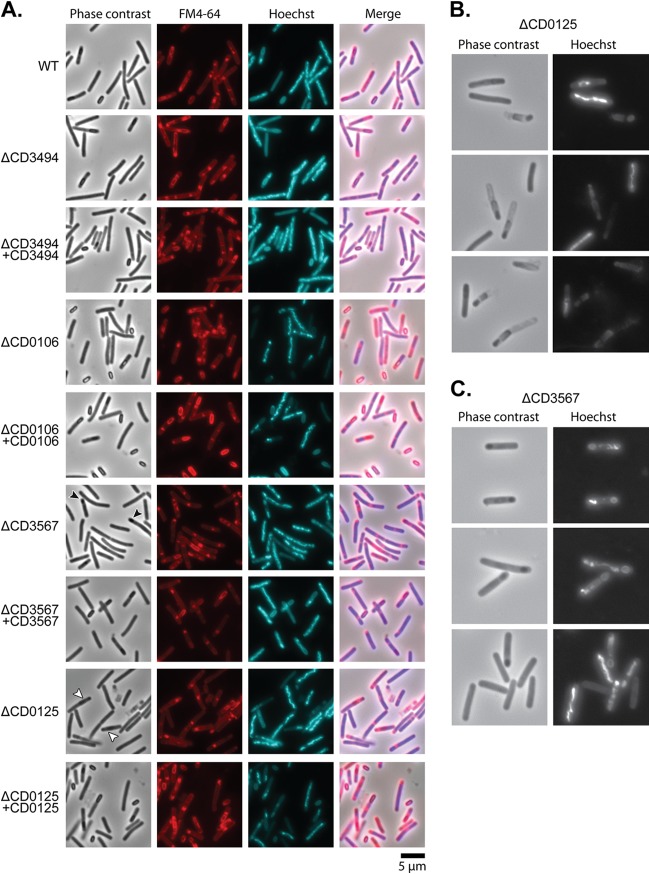
Microscopic examination of deletion mutants and complemented strains. (A) Cultures were sporulated on SMC agar for 24 h, harvested in PBS, and fluorescently labeled with a membrane stain (FM4-64) and DNA stain (Hoechst 33258). Both the ΔCD0106 and ΔCD3494 mutants appeared to produce normal phase-bright spores. The ΔCD0125 mutant failed to produce visible mature spores, and sporulation appeared to halt at forespore formation (white arrowheads). The ΔCD3567 mutant also did not produce mature spores, and only small, phase-dark spores were observed (black arrowheads). Complementation of the ΔCD0125 and ΔCD3567 mutants restored normal spore production. (B and C) Higher magnification micrographs of ΔCD0125 and ΔCD3567 mutants showing severe sporulation defects.

**FIG 4:**
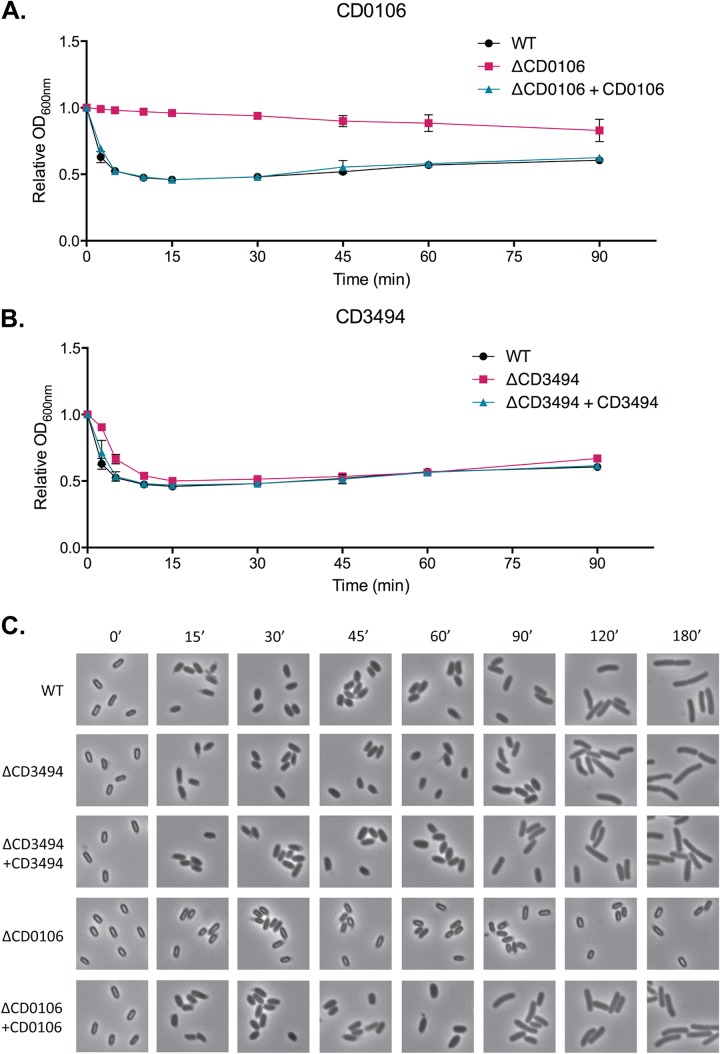
Germination efficiency of CD0106 and CD3494 isogenic deletion mutants. Spores of the parental strain 630Δ*erm* (WT), ΔCD0106, ΔCD3494, and complemented strains were produced on solid media and purified. (A and B) Purified spores were resuspended in BHIS supplemented with the germinant taurocholate (0.5%), and initiation of germination was monitored by measuring the drop in OD_600_ due to Ca^2+^-DPA release from the core. Data are expressed as the ratio of OD_600_ at each time point to *t*_0_. The ΔCD0106 mutant did not appear to germinate during the course of the experiment, and complementation restored germination to wild-type levels. The ΔCD3494 mutant appeared to germinate with a small delay compared to the parental strain but otherwise appeared to germinate normally. (C) Germinating suspensions were sampled, fixed in formaldehyde, and visualized by phase-contrast microscopy. In agreement with panel A, the ΔCD0106 mutant did not germinate in response to taurocholate, whereas the ΔCD3494 mutant appeared to germinate normally. Time (in minutes) is shown above the photos.

To confirm the role of each gene, four isogenic deletion strains were constructed in strain 630Δ*erm*, and each mutant was complemented by restoration of the native gene onto the chromosome by integration at the *pyrE* locus. No significant differences in growth rate were observed for the deletion strains compared to the parental strain 630Δ*erm* (see Fig. S3 in the supplemental material). The ability of each mutant to produce viable spores was then analyzed using a broth-based sporulation assay (Fig. 2), counting the total number of CFU and CFU following heat treatment (viable spores). Surprisingly, no significant decrease in sporulation efficiency was observed for the ΔCD630_34940 mutant despite a lack of transposon insertions in this gene in the 630Δ*erm* spore library. All remaining mutants produced significantly fewer viable spores than the wild-type strain. The ΔCD630_01060 and ΔCD630_01250 mutants produced 1,000-fold fewer spores than the wild type, and there was also an apparent delay in the initiation of sporulation in the ΔCD630_01060 mutant, with no spores detected before the 48-h time point. The phenotype of the ΔCD630_35670 mutant was even more severe, with no viable spores detected for the duration of the experiment. In each case, complementation at the *pyrE* locus fully restored sporulation to the wild-type level. In order to differentiate between defects in sporulation and germination, production of spores was followed using phase-contrast and fluorescence microscopy (Fig. 3). As predicted from the broth-based sporulation assay, the ΔCD630_34940 mutant appeared to sporulate as normal. In contrast, the ΔCD630_01250 and ΔCD630_35670 mutants both appeared to initiate spore morphogenesis, producing phase-dark immature spores, but neither mutant was capable of producing normal phase-bright mature spores. This phenotype has been previously described for an insertional disruption mutant of CD630_35670 (37). Interestingly, the ΔCD630_01060 mutant appeared to sporulate normally despite the dramatic decrease in viable spore numbers observed previously. This suggested that the ΔCD630_01060 mutant was defective in germination rather than sporulation. To examine this in greater detail, spores were purified, and taurocholate-induced germination was monitored by measuring the decrease in optical density resulting from Ca^2+^-dipicolinic acid (DPA) release and using phase-contrast microscopy (Fig. 4). The ΔCD630_01060 mutant showed no response to taurocholate induction, confirming a germination defect. Germination was restored to wild-type levels upon complementation with the CD630_01060 gene at the *pyrE* locus.

### Identification of essential genes in C. difficile R20291.

A higher-density transposon library was required to allow identification of essential genes with greater statistical power. The strain chosen was a representative of the clinically significant 027/NAP1 lineage, R20291, isolated during the first major ribotype 027 outbreak in the United Kingdom (38). In order to increase the insertion density, a total of approximately 750,000 erythromycin-resistant transposon mutants were pooled. TraDIS analysis of the library identified 77,256 unique insertion sites, an average of one insertion every 54 bp. Genes essential for growth *in vitro* under the conditions of this assay would be expected to have no transposon insertions in the library (e.g., Fig. 1D). To identify essential genes across the whole genome, an insertion index was calculated for each gene, normalizing the number of insertions to the length of the gene. A plot of these insertion indices displays a clear bimodal distribution, with the first, narrow peak indicating essential genes and the elongated second peak indicating genes that tolerate transposon insertions (Fig. 1C). Statistical analysis of these data (39) identified 404 essential genes and a further 33 genes that fell into the ambiguous region between essential and nonessential cutoffs (see Table S1 in the supplemental material). KEGG (Kyoto Encyclopedia of Genes and Genomes) analysis identified 25 pathways that were significantly enriched in essential genes (Table 1). As expected, these included genes involved in DNA replication (9 of 13 are essential genes) and mismatch repair (7 of 18 are essential genes), transcription (all 4 RNA polymerase subunits), and translation machinery (46 of 52 ribosomal genes), and many core metabolic pathways, including, for example, the genes required for tRNA biosynthesis (23 of 27 are essential genes), fatty acid biosynthesis (all 11 are essential genes), and peptidoglycan biosynthesis (12 of 19 are essential genes). The conserved core components of the Sec secretion machinery were also identified as essential, including the genes encoding both the housekeeping translocation ATPase SecA1 and the accessory ATPase SecA2. We have previously reported that *secA2* is essential (29) and identified the S-layer precursor as the major substrate of the C. difficile accessory Sec system. Interestingly, *slpA*, encoding the S-layer precursor, was also found to be essential in this screen, suggesting that the essentiality of the accessory Sec system may be due to the essentiality of its major substrate. No insertions were seen in a region of the genome encompassing R20291_2657 to R20291_2671, located downstream of the locus encoding *slpA* and other cell wall proteins. This cluster encodes many putative glycosyltransferases and suggests that C. difficile elaborates a surface polysaccharide that is required for cell growth. Intriguingly, 53 of the identified essential genes encode proteins with no obvious function based on homology, including 25 hypothetical proteins, 17 conserved hypothetical proteins, 8 hypothetical lipoproteins or membrane proteins, and 3 uncharacterized proteins.

**TABLE 1:** Pathway analysis of essential genes

KEGG pathway	No. of essential genes	Total no. of genes	Adjusted *P* value
Ribosomal	46	52	9.26 × 10^−37^
Aminoacyl-tRNA biosynthesis	23	27	3.00 × 10^−17^
Metabolic pathways	104	489	2.53 × 10^−11^
Fatty acid metabolism	12	16	6.41 × 10^−8^
Peptidoglycan biosynthesis	12	19	1.08 × 10^−6^
DNA replication	9	13	1.44 × 10^−5^
Purine metabolism	18	52	5.85 × 10^−5^
Terpenoid backbone biosynthesis	9	14	3.20 × 10^−4^
RNA polymerase	4	4	1.26 × 10^−3^
Protein export	7	13	1.8 × 10^−3^
Lysine biosynthesis	9	21	1.41 × 10^−3^
Pyrimidine metabolism	14	47	2.22 × 10^−3^
Homologous recombination	8	18	2.22 × 10^−3^
Secondary metabolite biosynthesis	37	200	5.11 × 10^−3^
Oxidative phosphorylation	8	21	6.33 × 10^−3^
Mismatch repair	7	18	1.07 × 10^−2^
Folate biosynthesis	5	11	2.08 × 10^−2^
Carbon metabolism	18	86	2.25 × 10^−2^
d-Glutamine and d-glutamate metabolism	3	4	2.25 × 10^−2^
Glycerophospholipid metabolism	6	16	2.27 × 10^−2^
Microbial metabolism in diverse environments	25	137	2.80 × 10^−2^
Biotin metabolism	4	8	2.80 × 10^−2^
Propanoate metabolism	6	17	2.81 × 10^−2^
Pantothenate and coenzyme A biosynthesis	5	13	3.51 × 10^−2^
Nicotinate and nicotinamide metabolism	4	9	4.09 × 10^−2^

### Identification of genes required for sporulation.

In order to identify genes required for sporulation from among the nonessential genes, the pooled R20291 library was grown overnight, sporulated on solid media, spores were purified on a HistoDenz gradient, and gDNA was extracted in duplicate from the overnight culture and pure spores. Transposon insertion sites were then identified using TraDIS and analyzed in comparison to the initial library. During production of spores from the initial transposon library, mutants could be lost through either selective or stochastic processes. To limit false discoveries, a stringent cutoff was applied to the analysis; only mutants with at least 20 reads present in both replicates were included. This cutoff limited the number of genes that could be assayed in each sample; of the 3,222 genes with transposon insertions in the initial library, 3,219 in the overnight culture samples and 3,168 in the spore samples satisfied these criteria and were assayable. Following overnight growth, only 392 genes showed statistically significant changes in the prevalence of the mutant at a false discovery rate (FDR) of 10%; however, in general, the magnitudes of these changes were not large, only 36 genes had a statistically significant absolute log_2_ fold change (log_2_FC) in the prevalence of mutants greater than 2 (Fig. 1E). In contrast, the prevalence of mutants in the purified spore samples indicated massive changes in the mutant population structure during spore formation. Fully 2,388 genes showed statistically significant changes in the prevalence of mutants over the experiment, 798 with a log_2_ fold change lower than or equal to −2, which suggests that they strongly affect sporulation (Fig. 1F; see Table S2 in the supplemental material).

Among the 798 genes required for sporulation, we identified *spo0A*, encoding the master regulator of sporulation, and *sigH*, encoding σ^H^, the key sigma factor of transition phase, both of which have been previously shown to be required for sporulation in C. difficile (9). Genes directly controlled by Spo0A and σ^H^ and thus involved in initiation of sporulation were also identified in this screen, including both the forespore and mother cell-specific early stage RNA polymerase sigma factors (σ^F^ and σ^E^, respectively) together with proteins controlling their activity (SpoIIAA, SpoIIAB, SpoIIE, and SpoIIGA). Many genes in the σ^F^ and σ^E^ regulons also appear to be required for sporulation, including genes involved in stage II (*spoIIQ*, *spoIID*, *spoIIP*, and *spoIIR*), stage III (*spoIIIAA*, *spoIIIAB*, *spoIIIAD*, *spoIIIAE*, *spoIIIAF*, *spoIIIAG*, *spoIIIAH*, and *spoIIID*), and stage IV (*spoIVA*, *spoIVB′*, and *sipL*) of sporulation. Among these genes, *spoIIR* has been previously characterized (12), and *spoIID*, *spoIIP*, *spoIIQ*, and genes belonging to the *spoIIIA* locus are of particular note, as they encode homologues of B. subtilis proteins involved in forespore engulfment. SpoIIID is a crucial component of the mother cell regulatory network, activating *sigK* expression and playing several auxiliary roles during spore morphogenesis (12, 40), while *spoIVA* and *sipL* encode key spore morphogenetic proteins involved in targeting coat proteins to the surface of the forespore (37). Even though little is known about the molecular mechanisms controlling the later steps in the signaling cascade regulating sporulation in C. difficile, a number of genes thought to be involved in these processes were identified in our screen. These genes included *sigG*, encoding the late-stage forespore-specific sigma factor σ^G^, and genes belonging to the σ^G^ regulon, *spoIVB*, *spoVAC*, *spoVAD*, and *spoVAE.* Other notable genes involved in sporulation were identified in this group, including genes encoding two small acid-soluble proteins, SspA and SspB, although the former did not meet our minimal fold change criterion (log_2_FC = 1.95; *Q* = 0.03).

**FIG 5:**
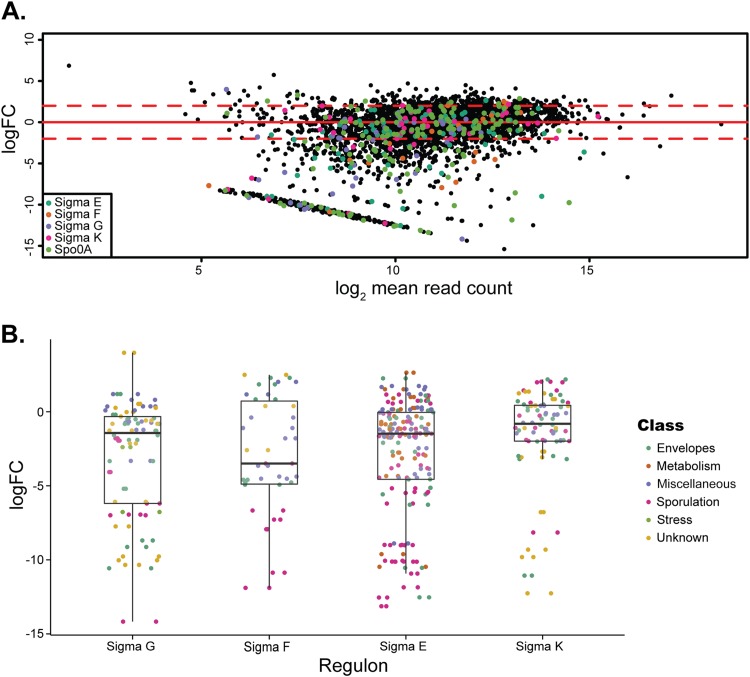
Comparison of genes required for sporulation with published regulons for Spo0A, σ^E^, σ^F^, σ^G^, and σ^K^. The 798 genes identified in our screen were cross-referenced with the previously identified regulons of Spo0A (41) and σ^E^, σ^F^, σ^G^, and σ^K^ (12). (A) Each gene is shown as a single point and colored according to the regulon; those genes not identified in the 5 published regulons are shown as black points. Dashed red lines indicate a log_2_FC of 2 and −2. (B) Log_2_FC in the spore transposon library of every gene previously identified as belonging to the σ^E^, σ^F^, σ^G^, and σ^K^ regulons colored by functional class, as defined in reference 12. The box-and-whisker plots show the interquartile range (IQR), with the median marked (black line). The whiskers indicate the highest/lowest point within 1.5× IQR of the upper/lower quartile.

Overall, Gene Set Enrichment Analysis (GSEA) of sporulation-related regulons determined in previous studies (12, 13, 41) revealed an overrepresentation of genes required for sporulation within the σ^E^, σ^F^, and σ^G^ regulons (Fig. 5A and B). However, as a whole, only the σ^F^ regulon showed a median log_2_FC value significantly below zero (Fig. 5B). Most genes belonging to the σ^K^ regulon, including those encoding spore coat proteins (CotA, CotCB, CotD, CotE, and CotF), exosporial glycoproteins (BclA1, BclA2, and BclA3), and the spore cortex lytic enzyme SleC, required for germination (42), showed little change in the number of insertions and clustered around a log_2_FC of 0. Despite this and the fact that a *sigK* mutant has been reported to produce low titers of heat-resistant spores devoid of the coat but with an apparently normal cortex layer (11, 40), the *sigK* gene itself was found to be required for sporulation. This could be due to the relatively low levels of sporulation of the mutant (4-log-unit drop in spore titers compared to that of the wild type) or loss of the aberrant spores during purification on density gradients. Interestingly, of the genes from the SigK regulon that were identified as having a strong effect on sporulation, the majority have no known function.

Other functional groups of genes that affect sporulation included genes encoding ABC transporters (57 genes), phosphotransferase system (37 genes), two-component systems (21 genes), and a large number of conserved hypothetical proteins (70 genes) and putative membrane proteins (54 genes). Importantly, the R20291 homologues of the two sporulation genes confirmed in the 630Δ*erm* mutant (CDR20291_0124 and CDR20291_3404) were also found to be required for normal sporulation (see Table S2 in the supplemental material).

## DISCUSSION

C. difficile is the main cause of antibiotic-associated diarrhea in nosocomial settings and is a significant cause of morbidity and mortality in Europe and the United States (1). Despite its importance as a human pathogen, our understanding of C. difficile lifestyle and virulence has lagged behind that of many other pathogenic bacteria due to a lack of genetic tools for the dissection of the organism. Recent advances in clostridial genetics (19, 20, 43) have led to key insights into many aspects of C. difficile biology but have also demonstrated that there are fundamental differences between C. difficile and other members of the *Firmicutes*, particularly in sporulation and germination. Here we have combined our recently developed tightly regulated inducible gene expression system (29) with a novel conditional plasmid origin to produce a *mariner* mutagenesis system suitable for generating saturating transposon libraries in C. difficile. We produced a proof-of-principle transposon library in the model strain 630Δ*erm* containing more than 44,000 unique mutants, confirming the utility of our mutagenesis system for high-throughput gene function analysis in C. difficile. In order to validate the library, we chose three genes predicted to play a role in sporulation and one gene with a role in germination for further study. Three of the isogenic mutants displayed the predicted phenotype; deletion of CD630_01250 or CD630_35670 resulted in severe sporulation defects, and a CD630_01060 mutant sporulated as normal but was incapable of germinating in response to the germinant taurocholate. Surprisingly, a CD630_34940 deletion mutant sporulated at levels equivalent to that of the parental strain despite clear differences in transposon insertion density in the gene before and after sporulation. CD630_34940 encodes a homologue of a B. subtilis spore coat-associated protein, YabP, and disruption of this gene would be expected to have an effect on coat assembly. CD630_34940 is the first gene in an apparent 3-gene operon CD630_34920 to CD630_34940. It is possible that the polar effects of a transposon insertion may explain the differences observed, although elucidating the exact role of this operon in sporulation will require further study.

Although confirming the validity of our transposon mutagenesis approach, the size and density of the 630Δ*erm* library was insufficient to perform a statistical analysis of gene essentiality. However, our method is readily scalable. Rather than attempt to generate a saturating library in the same genetic background, we chose to focus on the most clinically important C. difficile lineage currently in circulation, ribotype 027 (BI and NAP1 [38]). We generated a transposon library containing 77,256 unique mutants in strain R20291. TraDIS analysis of this library identified 404 essential genes, 11% of the annotated genes, and a further 33 genes that fell in an ambiguous area between essential and nonessential gene cutoffs. This is of the same order of magnitude as in other organisms; for example; recent studies identified 505 essential genes in Burkholderia pseudomallei out of a total of 5,942 genes (8.5% [44]) and 353 essential genes in *Salmonella enterica* serovar Typhimurium out of a total of 4,530 (7.8% [39]). As expected, the list of essential genes in C. difficile includes those known to be involved in central cellular processes, such as DNA replication and repair, transcription, translation, protein secretion, cell envelope biogenesis, cell division, and many core metabolic pathways. Although not surprising, identification of these genes serves to validate our transposon screen. Of greater interest will be the 53 essential genes that have no known or predicted function, as these potentially represent attractive targets for future species-specific therapeutics. Of the 351 essential genes with functions predicted from homology, most have not been experimentally confirmed in C. difficile, and for many, the cellular processes they contribute to are completely unknown. This is the first time a high-throughput forward genetic screen has been carried out in C. difficile, and these data should form an invaluable resource for the community.

Sporulation is an ancient and highly complex cell differentiation process that has been studied in great detail in B. subtilis (45). As an obligate anaerobe, the process of sporulation is absolutely critical for the transmission of C. difficile from one host to the next (5). Despite overlaps in the regulatory cascades leading to spore formation, there are significant differences in C. difficile, including the mechanism of cascade activation via Spo0A (10) and the order and timing of sigma factor activation (11, 13). The spore structural proteins are also poorly conserved between species (46). We have identified 798 genes, mutation of which was strongly associated with a sporulation defect, either by direct disruption of the sporulation process or indirectly due to a more general fitness defect that results in disrupted or delayed sporulation. Only a small number of these genes have been studied in C. difficile, but several have well-characterized homologues in other spore-forming species, including B. subtilis. The subset of genes with known or predicted roles in sporulation includes the master regulator *spo0A* gene and the four sporulation-specific sigma factor genes *sigE*, *sigF*, *sigG*, and *sigK*. Of greater interest are the large number of genes that have no previously characterized role in sporulation. The vast majority of these have not been studied in C. difficile; indeed; fully 334 genes have gene ontology terms including the word “putative,” 68 have “conserved hypothetical,” 28 have “hypothetical,” and 19 have “uncharacterized.” Also of interest are the large number of identified genes encoding ABC transporters, the phosphotransferase system, and two-component systems. Considerable analysis will be required to elucidate the roles of these genes in sporulation, but the data presented here should act as a road map for future research into C. difficile sporulation.

One of the greatest challenges in the clinical management of *C. difficile* infection (CDI) is our reliance on antibiotic therapy for treating the infection. As CDI is essentially a consequence of dysbiosis, treatment with broad-spectrum antibiotics, such as metronidazole or vancomycin, leaves the patient acutely susceptible to reinfection, and as a result, relapse is common (47). It would be preferable to treat infection with narrow-spectrum, or even species-specific, therapeutics to avoid disturbance of the intestinal microflora. To develop such therapeutics, we need a detailed understanding of the differences between C. difficile and other species, particularly in sporulation, and also of the complement of essential genes, as their products are attractive targets for therapeutic intervention.

## MATERIALS AND METHODS

### Strains and growth conditions.

C. difficile strains 630Δ*erm* (ribotype 012) and R20291 (ribotype 027) were routinely grown on BHI agar or in TY broth without thioglycolate (48), except where otherwise stated. Escherichia coli strains NEB5α (New England Biolabs) and CA434 (HB101 carrying R702) were grown on LB agar or in LB broth (VWR). Cultures were supplemented with thiamphenicol (15 µg/ml), erythromycin (5 µg/ml), lincomycin (20 µg/ml), chloramphenicol (15 µg/ml), taurocholate (0.5%), or anhydrotetracycline (100 ng/ml) where appropriate.

### Construction of a *mariner* delivery plasmid for C. difficile.

The *Himar1 mariner* transposase gene was synthesized and cloned into a C. difficile-E. coli shuttle vector, yielding pMar1. The gene was subcloned into the C. difficile-E. coli shuttle plasmid pRPF177 (29) between the SacI and BamHI sites, placing expression of the transposase under the control of the tetracycline-inducible P_tet_ promoter. The resulting plasmid, pRPF213, was further modified by the addition of an *ermB*-based transposon. The *ermB* gene with its native promoter was amplified from plasmid pMTL82254 (43), a transcriptional terminator (from the *fdx* gene) was added downstream of *ermB*, and *mariner* inverted terminal repeats (ITRs) were added to each end. The resulting transposon was cloned into the BstXI site of pRPF213, yielding the final *mariner* delivery plasmid pRPF215. A transposition negative-control plasmid, pRPF222, was constructed by deleting the nucleotides corresponding to residues 244 to 291 of the *Himar1* transposase. The resulting plasmid retains the functional *ermB* marker, but the transposon should be incapable of transferring to the chromosome.

### Transposon mutagenesis of C. difficile.

Cultures of C. difficile carrying pRPF215 were grown overnight in TY broth supplemented with thiamphenicol, subcultured to an optical density at 600 nm (OD_600_) of 0.05, and grown to an OD_600_ of 0.3. The mid-logarithmic-phase cultures were then spread on BHIS (31) agar plates containing anhydrotetracycline and erythromycin (strain 630Δ*erm*) or lincomycin (strain R20291). After 18-h growth, each colony (approximately 85,000 for strain 630Δ*erm* and approximately 750,000 for strain R20291) represented a unique transposition event. All colonies were pooled and resuspended in TY broth supplemented with anhydrotetracycline and erythromycin or lincomycin to produce the initial transposon mutant library. Samples were harvested for DNA extraction (see below). The pooled library was then sporulated as described previously (49). Briefly, the pooled library was subcultured to an OD_600_ of 0.1 in TGY broth (49) supplemented with anhydrotetracycline and erythromycin/lincomycin and grown overnight. Samples were harvested for DNA extraction. The TGY culture was then diluted 1:10 in SMC broth, (49) grown to an OD_600_ of 0.6, and then spread on SMC agar plates. After a week on SMC agar, spores were recovered in H_2_O and purified on a 20 to 50% HistoDenz (Sigma-Aldrich) gradient (50). Spores from the 630Δ*erm* library were germinated in BHIS broth supplemented with erythromycin and taurocholate (50). Samples of the TGY overnight culture, spores, and germinated library were retained for DNA extraction.

### DNA extraction.

For extraction of genomic DNA (gDNA) from vegetative cells, frozen bacterial samples were thawed and resuspended in 2-ml lysis buffer (200 mM NaCl, 50 mM EDTA, 20 mM Tris-HCl [pH 8.0], 2 mg/ml lysozyme) and incubated at 37°C for 1 h. Lysed bacterial samples were then sequentially treated with pronase (0.5 mg/ml; 55°C overnight), *N*-lauroylsarcosine (2%; 1 h at 37°C) and RNase (0.05 mg/ml; 1 h at 37°C). Samples were then extracted twice with phenol-chloroform-isoamyl alcohol (25:24:1) and twice with chloroform-isoamyl alcohol (24:1) using heavy phase lock gel tubes (5 Prime). gDNA was then precipitated with ethanol overnight at −20°C, washed with 70% ethanol, and finally resuspended in 50 µl of nuclease-free water. For extraction of gDNA from C. difficile spores, samples were resuspended in lysis buffer as described above and then broken over glass beads using a FastPrep-24 instrument (MP Biomedicals) at 6.5 m/s for three 45-s cycles, with cooling on ice between cycles. DNA was then purified as described above.

### Illumina sequencing and analysis.

gDNA was extracted, fragmented, and sequenced on a Hiseq2500 or Hiseq2000 Illumina platform, generating approximately 2 million 50-bp single-end reads per sample. The resulting FASTQ files were filtered for 10 bases matching the 3′ end of the transposon. These transposon tags were stripped from the resulting reads, and the stripped reads were then mapped to their corresponding reference sequences using SMALT-0.7.2. The precise insertion site of the transposon was determined using the Bio::Tradis tools (https://github.com/sanger-pathogens/Bio-Tradis). Insertion sites and read counts were tabulated per gene, and further analysis was conducted using R.

Gene essentiality was assayed as described previously (39). Briefly, an insertion index (number of insertion sites/gene length) was calculated for each gene. The observed distribution of insertion indices was bimodal, and gamma distributions were fitted to the putative essential (mode at 0) and nonessential peaks of the empirical distribution. Log_2_ likelihood ratios (LLR) were calculated between the fitted distributions, and a gene was classified as essential if it had an LLR of less than −2, leading to an essentiality cutoff at an insertion index of 0.0015. Similarly, a gene was classified as nonessential if it had an LLR of >2, giving an insertion index cutoff of 0.0021. Insertion indices falling between these two values were classified as “ambiguous.” Enrichment of essential genes in KEGG (Kyoto Encyclopedia of Genes and Genomes) pathways (51) was determined using a hypergeometric test.

To identify genes important for overnight growth and sporulation, edgeR (52) was used to identify significant differences in read counts over genes before and after selection. The TMM (trimmed mean of M values) normalization was applied, and tagwise dispersion was estimated. Only genes exhibiting greater than 20 reads in both replicates of at least one of the conditions being assayed were tested for differences in the prevalence of mutants. *P* values were corrected for multiple testing by the Benjamini-Hochberg method, and genes with a corrected *P* value (*Q* value) of <0.1 (a hypothetical 10% false discovery rate [FDR]) and an absolute log fold change (logFC) of >2 were considered significant. To determine enrichment of sporulation-related regulons, we used transcriptomic data from previous publications (Spo0A [41] and σ^E^, σ^F^, σ^G^, and σ^K^ [12]) as gene sets for Gene Set Enrichment Analysis (GSEA) analysis (53). GSEA version 2.1.0 was run in ranked-list mode with enrichment statistic “weighted” on the per-gene logFCs calculated by edgeR.

### Construction of individual deletion mutants and complementation.

In-frame deletions of individual genes were generated in C. difficile strain 630Δ*erm*Δ*pyrE* as described previously (20). Briefly, homologous recombination plasmids were generated by adding appropriate 750-bp upstream and downstream homology arms to pMTL-YN3 using Gibson assembly. The resulting plasmids were conjugated into strain 630Δ*erm*Δ*pyrE*, and deletion mutants were isolated as described previously (20). Putative mutants were confirmed by PCR. The *pyrE* gene was subsequently restored using plasmid pMTL-YN1 as described previously (20). Each mutant was complemented on the chromosome by cloning a fragment containing the wild-type gene with its native promoter (identified using BPROM [SoftBerry]) into pMTL-YN1C for integration into the *pyrE* locus as described previously (20).

### Sporulation and germination efficiency.

Overnight cultures of C. difficile in BHIS broth were diluted to an OD_600_ of 0.01 in fresh broth, grown to an OD_600_ of approximately 0.6, and diluted again to an OD_600_ of 0.0001 before growth overnight to stationary phase. This allowed synchronization of growth and minimized the carryover of spores from initial cultures. The relative proportions of vegetative cells and spores were then monitored at 24-h intervals for 5 days. At each time point, the total number of bacteria was determined by counting the number of CFU on BHIS agar supplemented with 0.1% taurocholate. To determine the number of spores, samples were incubated at 70°C for 30 min, prior to counting CFU.

To determine germination efficiency, purified spores were resuspended to an OD_600_ of 1.0 in 10 ml BHIS supplemented with 0.5% taurocholate. Spore germination was determined by monitoring the changes in OD_600_ due to Ca^2+^-dipicolinic acid (DPA) release and outgrowth. Data are expressed as the ratio of OD_600_ at each time point to time zero (*t*_0_). Germination was also monitored by phase-contrast microscopy.

### Microscopy.

One milliliter of culture was stained with FM4-64 membrane dye at a concentration of 0.5 µg/ml, centrifuged at 5,000 × *g* for 10 min at 4°C, and washed with 1 ml of phosphate-buffered saline (PBS). DNA staining was carried out by adding Hoechst 33258 dye to the mounting medium to a final concentration of 20 µg/ml. Images were captured using an Eclipse E600 microscope (Nikon) with a Retiga-400R charge-coupled device (CCD) (Q-Imaging).

### Nucleotide sequence accession numbers.

The GenBank accession numbers for the raw sequencing data for the C. difficile 630Δ*erm* transposon library are ERR245853 to ERR245855 and ERR237766, and those for the R20291 transposon library are ERR377408 to ERR377413 and ERR377416 to ERR377421 (see Table S3 in the supplemental material).

## SUPPLEMENTAL MATERIAL

Figure S1  A *mariner* delivery vector for C. difficile. (A) The conditional plasmid pRPF177 was used as the basis for the construction of a *mariner* delivery vector. The *gusA* gene between SacI and BamHI was replaced with a codon-optimized gene encoding the *Himar1* transposase, placing expression under the control of the inducible P_tet_ promoter. An *ermB* transposon was then constructed by successive rounds of PCR to add a 3′ transcriptional terminator and *mariner* inverted terminal repeats (ITRs) and cloned into the BstXI site. (B) The resulting *mariner* delivery vector is pRPF215. (C and D) Colony morphology of C. difficile 630Δ*erm* on BHIS agar supplemented with erythromycin and with anhydrotetracycline induction (D) or without anhydrotetracycline induction (C). Induction of transposition results in a wide range of unusual colony morphologies. Download Figure S1, TIF file, 1.8 MB

Figure S2  Selection of candidate genes for further analysis. (A) Following sporulation on solid media, all growth was harvested in PBS (left-hand panel) and spores were purified on a HistoDenz density gradient (right-hand panel). (B) TraDIS data from the initial transposon library, spores, and following germination were examined manually. Four candidate genes were chosen for further analysis. Each had multiple insertions in the initial library but displayed large differences following sporulation or germination. Each vertical line represents a unique transposon insertion site, with height indicating the total number of Illumina reads at that point. Download Figure S2, TIF file, 1.9 MB

Figure S3  Growth profiles of isogenic C. difficile 630Δ*erm* mutants. Overnight cultures were subcultured to an OD_600_ of 0.05 in fresh TY broth, and growth was monitored for 9 h. No significant differences were observed for any mutant or complemented mutant in comparison to the 630Δ*erm* parental strain. Displayed are the means and standard deviations from two biological replicates. Download Figure S3, TIF file, 1 MB

Table S1  Genes required for *in vitro* growth of C. difficile R20291 Table S1, PDF file, 0.1 MB

Table S2  Genes required for sporulation in C. difficile R20291 Table S2, PDF file, 0.1 MB

Table S3  Sequencing data accession numbers Table S3, DOCX file, 0.1 MB
