# Supplementary figures and images for: High-Throughput Analysis of Gene Essentiality and Sporulation in Clostridium difficile
Source: mBio. 2015 Feb 24;6(2):e02383-14. doi: 10.1128/mBio.02383-14 (PMC4358009; doi:10.1128/mBio.02383-14)

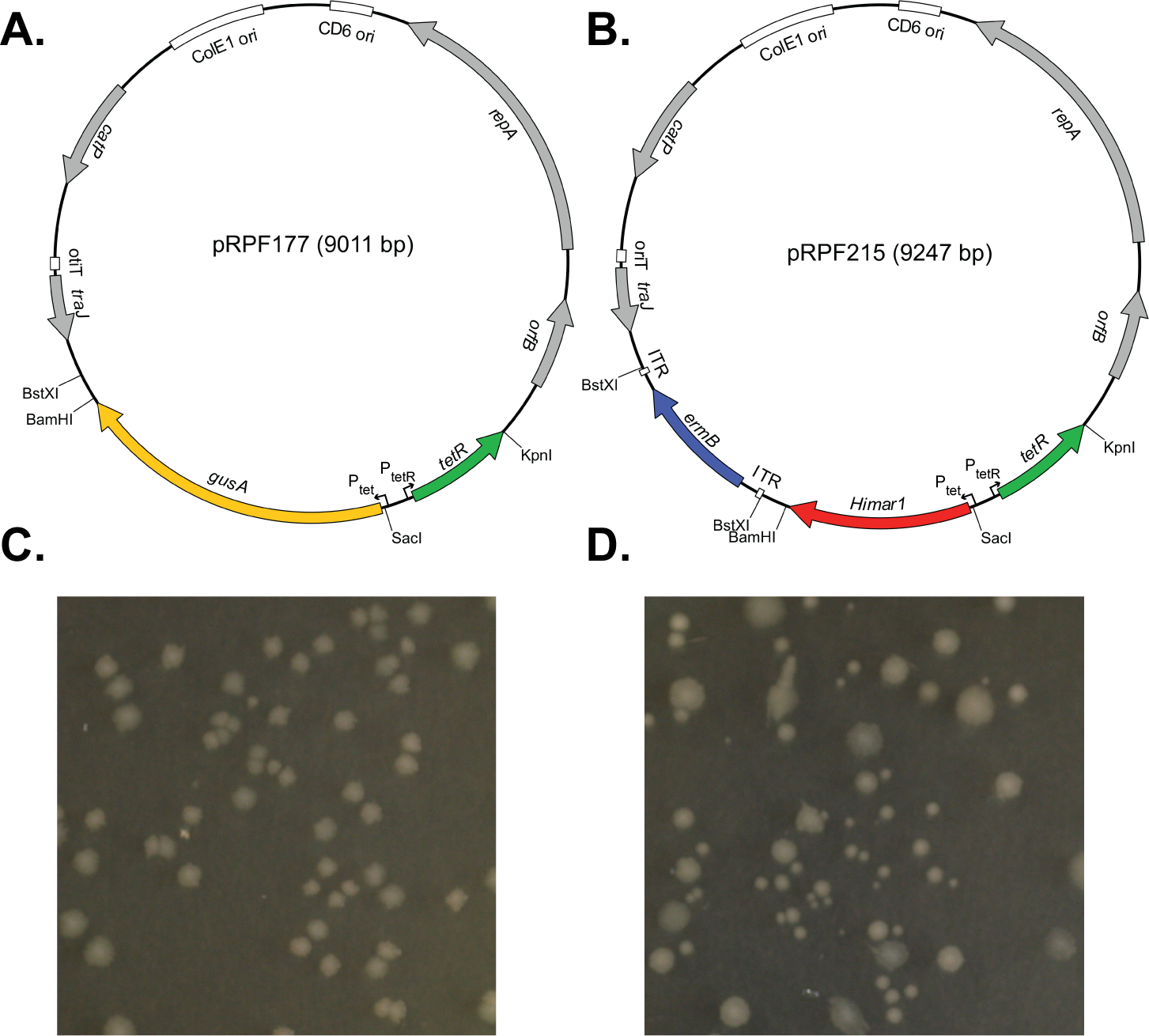

Supplement: Figure S1 — A mariner delivery vector for C. difficile. (A) The conditional plasmid pRPF177 was used as the basis for the construction of a mariner delivery vector. The gusA gene between SacI and BamHI was replaced with a codon-optimized gene encoding the Himar1 transposase, placing expression under the control of the inducible Ptet promoter. An ermB transposon was then constructed by successive rounds of PCR to add a 3′ transcriptional terminator and mariner inverted terminal repeats (ITRs) and cloned into the BstXI site. (B) The resulting mariner delivery vector is pRPF215. (C and D) Colony morphology of C. difficile 630Δerm on BHIS agar supplemented with erythromycin and with anhydrotetracycline induction (D) or without anhydrotetracycline induction (C). Induction of transposition results in a wide range of unusual colony morphologies. Download [file mbo001152196sf1.tif]

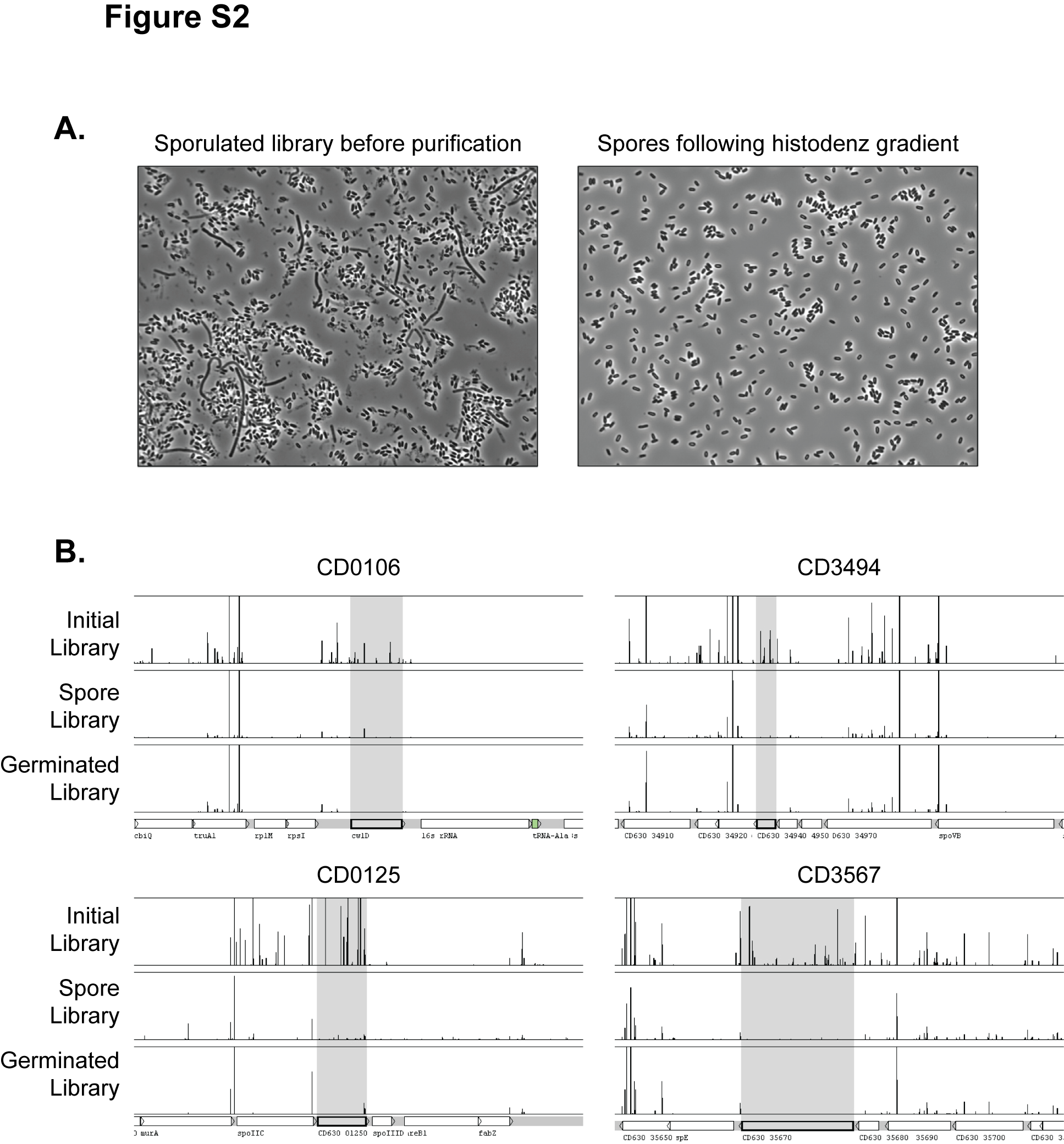

Supplement: Figure S2 — Selection of candidate genes for further analysis. (A) Following sporulation on solid media, all growth was harvested in PBS (left-hand panel) and spores were purified on a HistoDenz density gradient (right-hand panel). (B) TraDIS data from the initial transposon library, spores, and following germination were examined manually. Four candidate genes were chosen for further analysis. Each had multiple insertions in the initial library but displayed large differences following sporulation or germination. Each vertical line represents a unique transposon insertion site, with height indicating the total number of Illumina reads at that point. Download [file mbo001152196sf2.tif]

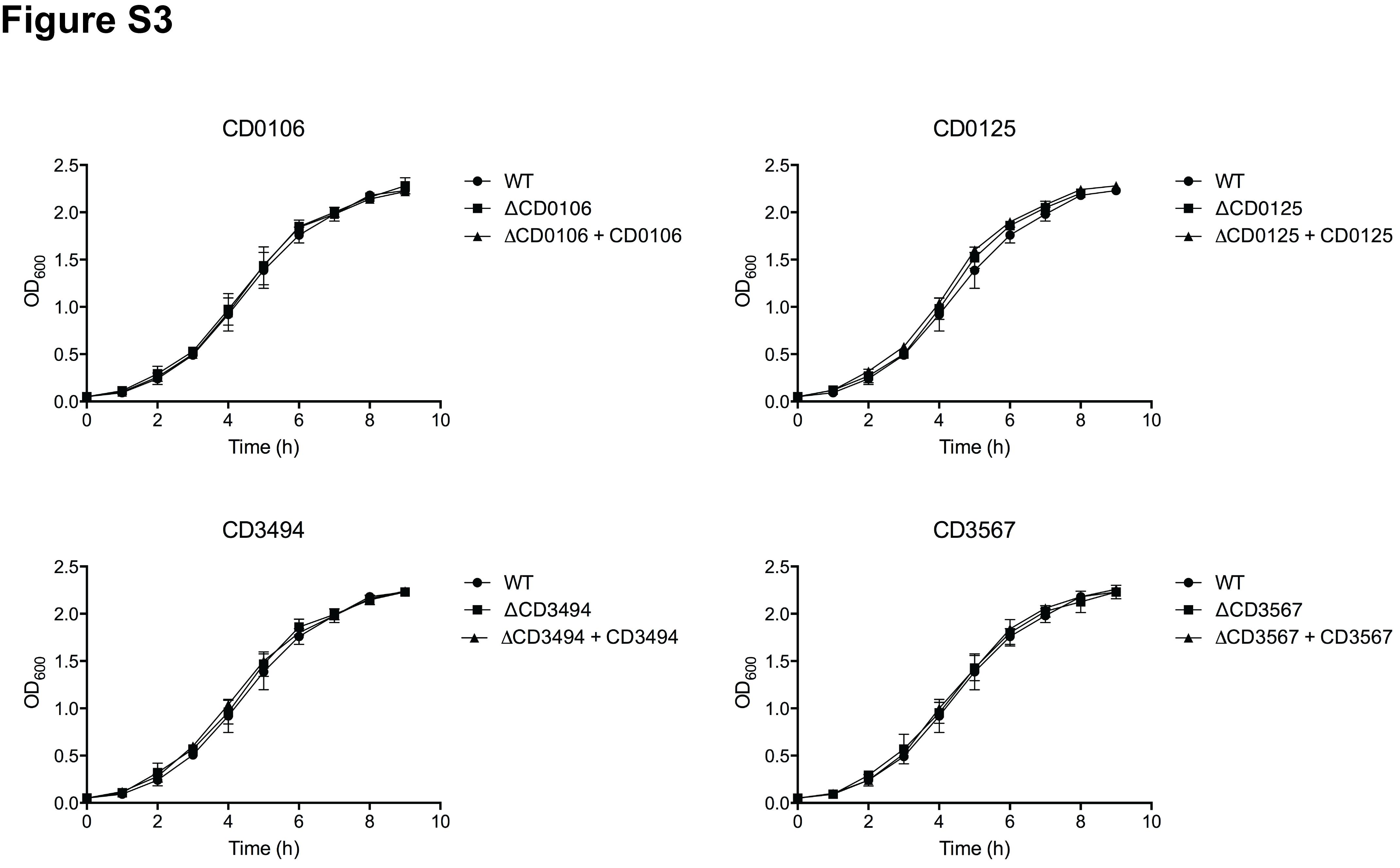

Supplement: Figure S3 — Growth profiles of isogenic C. difficile 630Δerm mutants. Overnight cultures were subcultured to an OD600 of 0.05 in fresh TY broth, and growth was monitored for 9 h. No significant differences were observed for any mutant or complemented mutant in comparison to the 630Δerm parental strain. Displayed are the means and standard deviations from two biological replicates. Download [file mbo001152196sf3.tif]
