# Supplementary material for: High-Throughput Analysis of Gene Essentiality and Sporulation in Clostridium difficile
Source: mBio. 2015 Feb 24;6(2):e02383-14. doi: 10.1128/mBio.02383-14 (PMC4358009; doi:10.1128/mBio.02383-14)
Supplement: Table S1 — Genes required for in vitro growth of C. difficile R20291 [file mbo001152196st1.pdf]

Ambiguous

locus_tag	Insertion Count	Insertion Index	Gene Length (bp)	Annotated Function
CDR20291_0100	1	0.001567398	708	ABC transporter
CDR20291_0370	2	0.001958864	1134	acyl-CoA dehydrogenase
CDR20291_0646	1	0.001754386	633	methenyltetrahydrofolate cyclohydrolase
CDR20291_0840	1	0.00154321	720	putative phosphoesterase
CDR20291_0959	2	0.001984127	1119	transposase-like protein b
CDR20291_0975	1	0.001949318	570	electron transport complex protein
CDR20291_1323	1	0.002028398	547	putative ruberythrin
CDR20291_1342	4	0.00162206	2739	putative two-component sensor histidine kinase
CDR20291_1416	1	0.001567398	708	hypothetical protein
CDR20291_1709	1	0.001730104	642	putative phosphoglycerate mutase
CDR20291_1711	1	0.001706485	651	cytidylate kinase
CDR20291_1770	2	0.001638002	1356	hypothetical protein
CDR20291_1800	2	0.001657001	1341	putative conjugative transposon mobilization protein
CDR20291_1836	1	0.001697793	654	putative ethanolamine/propanediol utilization protein
CDR20291_1842	1	0.001529052	726	putative ethanolamine utilization/propanediol conserved hypothetical protein
CDR20291_1852	1	0.001754386	633	putative membrane protein
CDR20291_1870	1	0.001587302	699	ABC transporter
CDR20291_2203	1	0.001517451	732	conserved hypothetical protein
CDR20291_2353	2	0.001923077	1155	chaperone protein
CDR20291_2359	2	0.001572327	1413	putative poly(A) polymerase
CDR20291_2444	1	0.002	555	rna methyltransferase
CDR20291_2461	1	0.001652893	672	ribulose-phosphate 3-epimerase
CDR20291_2520	2	0.001675042	1326	putative GTP-binding protein
CDR20291_2521	2	0.001657001	1341	putative fes-containing cyanobacterial-specific oxidoreductase
CDR20291_2667	2	0.002068252	1074	putative mannose-1-phosphate guanylyltransferase
CDR20291_2695	1	0.001841621	603	holliday junction DNA helicase
CDR20291_2716	2	0.001986097	1118	transposase-like protein B
CDR20291_2950	2	0.001984127	1119	transposase-like protein b

CDR20291_3090	3	0.002031144	1641	exosporium glycoprotein
CDR20291_3166	1	0.001897533	585	ATP-dependent Clp protease proteolytic subunit
CDR20291_3223	1	0.00155521	714	putative phosphoesterase
CDR20291_3472	1	0.001587302	699	putative abc transporter
CDR20291_3478	2	0.001984127	1119	transposase-like protein b

Essential Genes

locus_tag	Insertion Count	Insertion Index	Gene Length (bp)	Annotated Function
CDR20291_0003	1	0.000873362	1272	seryl-tRNA synthetase
CDR20291_0004A	0	0	186	hypothetical protein
CDR20291_0005	0	0	1638	DNA polymerase III subunit gamma/tau
CDR20291_0036	0	0	747	2-C-methyl-D-erythritol 4-phosphate cytidylyltransferase
CDR20291_0037	0	0	486	2-C-methyl-D-erythritol 2
CDR20291_0038	1	0.000647249	1716	prolyl-tRNA synthetase
CDR20291_0040	0	0	1482	glutamyl-tRNA synthetase
CDR20291_0041	1	0.000791139	1404	cysteinyl-tRNA synthetase
CDR20291_0043	0	0	831	thymidylate synthase
CDR20291_0051	1	0.000932836	1191	elongation factor TU
CDR20291_0052	0	0	150	50S ribosomal protein L33
CDR20291_0053	0	0	222	preprotein translocase SecE subunit
CDR20291_0054	0	0	543	transcription antitermination protein
CDR20291_0055	0	0	426	50S ribosomal protein L11
CDR20291_0056	0	0	699	50S ribosomal protein L1
CDR20291_0057	0	0	507	50S ribosomal protein L10
CDR20291_0060	0	0	3717	DNA-directed RNA polymerase beta chain
CDR20291_0061	1	0.000318674	3486	DNA-directed RNA polymerase beta' chain
CDR20291_0062	0	0	426	30S ribosomal protein S12
CDR20291_0063	0	0	471	30S ribosomal protein S7
CDR20291_0064	1	0.000537346	2067	translation elongation factor G
CDR20291_0065	0	0	1192	elongation factor TU
CDR20291_0067	0	0	630	50S ribosomal protein L3
CDR20291_0068	0	0	654	50S ribosomal protein L4
CDR20291_0069	0	0	291	50S ribosomal protein L23
CDR20291_0070	0	0	831	50S ribosomal protein L2
CDR20291_0071	0	0	282	30S ribosomal protein S19

CDR20291_0072	0	0	336	50S ribosomal protein L22
CDR20291_0073	0	0	816	30S ribosomal protein S3
CDR20291_0074	0	0	432	50S ribosomal protein L16
CDR20291_0075	0	0	204	50S ribosomal protein L29
CDR20291_0076	0	0	255	30S ribosomal protein S17
CDR20291_0078	0	0	309	50S ribosomal protein L24
CDR20291_0079	0	0	543	50S ribosomal protein L5
CDR20291_0080	0	0	186	30S ribosomal protein S14
CDR20291_0081	0	0	399	30S ribosomal protein S8
CDR20291_0082	0	0	543	50S ribosomal protein L6
CDR20291_0083	0	0	369	50S ribosomal protein L18
CDR20291_0084	0	0	510	30S ribosomal protein S5
CDR20291_0085	0	0	186	50S ribosomal protein L30
CDR20291_0086	0	0	444	50S ribosomal protein L15
CDR20291_0087	0	0	1269	preprotein translocase SecY subunit
CDR20291_0088	0	0	666	adenylate kinase
CDR20291_0089	0	0	747	methionine aminopeptidase
CDR20291_0090	0	0	279	putative ribosomal protein
CDR20291_0091	0	0	219	translation initiation factor IF-1
CDR20291_0092	0	0	114	50S ribosomal protein L36
CDR20291_0093	0	0	372	30S ribosomal protein S13
CDR20291_0094	0	0	399	30S ribosomal protein S11
CDR20291_0095	0	0	624	30S ribosomal protein S4
CDR20291_0096	0	0	948	DNA-directed RNA polymerase alpha chain
CDR20291_0097	0	0	342	50S ribosomal protein L17
CDR20291_0102	0	0	343	50S ribosomal protein L13
CDR20291_0103	0	0	393	30S ribosomal protein S9
CDR20291_0106	1	0.000472367	2352	anaerobic ribonucleoside-triphosphate reductase
CDR20291_0107	0	0	540	anaerobic ribonucleoside-triphosphate reductase activating protein
CDR20291_0110	0	0	120	conserved hypothetical protein

CDR20291_0118	0	0	1407	phosphoglucomutase/phosphomannomutase mutase
CDR20291_0119	0	0	1833	glucosamine--fructose-6-phosphate aminotransferase
CDR20291_0122	1	0.000873362	1272	UDP-N-acetylglucosamine 1-carboxyvinyltransferase 1
CDR20291_0126	1	0.001089325	1020	rod shape-determining protein
CDR20291_0127	0	0	429	(3R)-hydroxymyristoyl-[acyl carrier protein] dehydratase
CDR20291_0129	0	0	1194	S-adenosylmethionine synthetase
CDR20291_0142	1	0.00041511	2676	preprotein translocase SecA subunit
CDR20291_0143	1	0.001091703	1017	peptide chain release factor 2
CDR20291_0149	0	0	702	putative glycoprotease
CDR20291_0150	0	0	429	putative ribosomal-protein-alanine acetyltransferase
CDR20291_0173	0	0	168	ferredoxin
CDR20291_0194	0	0	285	10 kDa chaperonin
CDR20291_0195	1	0.000681663	1629	60 kDa chaperonin
CDR20291_0198	1	0.000723066	1536	GMP synthase
CDR20291_0338	0	0	1599	manganese-dependent inorganic pyrophosphatase
CDR20291_0351	0	0	693	putative phosphoesterase
CDR20291_0374	0	0	927	putative fructose-bisphosphate aldolase
CDR20291_0377	0	0	1119	transposase-like protein b
CDR20291_0382	1	0.001234568	900	integrase
CDR20291_0405A	0	0	168	hypothetical protein
CDR20291_0447A	0	0	153	conserved hypothetical protein
CDR20291_0472	0	0	360	putative penicillin-binding protein repressor
CDR20291_0481	1	0.001468429	756	putative endonuclease
CDR20291_0498	1	0.000551876	2013	threonyl-tRNA synthetase
CDR20291_0500	0	0	1119	transposase-like protein b
CDR20291_0526	0	0	201	putative membrane protein
CDR20291_0554A	0	0	240	conserved hypothetical protein
CDR20291_0581	0	0	555	putative transcriptional regulator
CDR20291_0611	0	0	423	translation initiation factor IF-3
CDR20291_0612	0	0	195	50S ribosomal protein L35

CDR20291_0613	0	0	357	50S ribosomal protein L20
CDR20291_0622	0	0	1335	probable cation transport protein
CDR20291_0623	0	0	672	putative cation transport protein
CDR20291_0625	1	0.001078749	1029	phenylalanyl-tRNA synthetase alpha chain
CDR20291_0626	0	0	2394	phenylalanyl-tRNA synthetase beta chain
CDR20291_0627	0	0	609	putative cell-division protein
CDR20291_0637	0	0	1701	arginyl-tRNA synthetase
CDR20291_0645	1	0.000662252	1677	formate--tetrahydrofolate ligase
CDR20291_0647	1	0.001272265	873	putative FolD bifunctional protein [includes: methylenetetrahydrofolate de
CDR20291_0697	1	0.000992063	1119	transposase-like protein b
CDR20291_0712	0	0	2694	penicillin-binding protein
CDR20291_0725	1	0.001468429	756	NH3-dependent NAD(+) synthetase
CDR20291_0809	0	0	1119	transposase-like protein B
CDR20291_0828	0	0	1506	conserved hypothetical protein
CDR20291_0868	0	0	483	putative membrane protein
CDR20291_0896	4	0.00128123	3468	ATP-dependent nuclease subunit B
CDR20291_0905	0	0	801	putative inorganic polyphosphate/ATP-NAD kinase
CDR20291_0965	0	0	603	putative dephospho-CoA kinase
CDR20291_0980	0	0	585	septum formation protein
CDR20291_0981	0	0	660	DNA repair protein
CDR20291_0982	0	0	1062	rod shape-determining protein
CDR20291_0983	0	0	894	putative rod shape-determining protein
CDR20291_0984	0	0	489	putative membrane protein
CDR20291_0985	2	0.000745712	2979	putative penicillin-binding protein
CDR20291_0986	0	0	684	septum site-determining protein
CDR20291_0987	0	0	798	septum site-determining protein (cell division inhibitor)
CDR20291_0988	0	0	285	cell division topological specificity factor
CDR20291_0989	0	0	1131	rod shape-determining protein
CDR20291_0998	0	0	312	50S ribosomal protein L21
CDR20291_1000	0	0	291	50S ribosomal protein L27

CDR20291_1001	0	0	1287	Spo0B-associated GTP-binding protein
CDR20291_1014	0	0	174	50S ribosomal protein L32
CDR20291_1015	0	0	558	DeoR-family transcriptional regulator (fatty acid and phospholipid biosynth
CDR20291_1016	0	0	1023	fatty acid/phospholipid synthesis protein
CDR20291_1017	0	0	987	3-oxoacyl-[acyl-carrier-protein] synthase III
CDR20291_1018	0	0	930	trans-2-enoyl-ACP reductase
CDR20291_1019	1	0.001168224	951	malonyl coa-acyl carrier protein transacylase
CDR20291_1020	0	0	750	3-oxoacyl-[acyl-carrier protein] reductase
CDR20291_1021	0	0	225	acyl carrier protein
CDR20291_1022	0	0	1239	3-oxoacyl-[acyl-carrier-protein] synthase II
CDR20291_1025	0	0	249	conserved hypothetical protein
CDR20291_1032	0	0	213	stage III sporulation protein AC
CDR20291_1038	0	0	363	conserved hypothetical protein
CDR20291_1044	0	0	438	putative membrane protein
CDR20291_1053	0	0	1122	putative pyrophosphokinase
CDR20291_1054	1	0.001267427	876	putative exported protein
CDR20291_1055	0	0	693	putative glycosyltransferase
CDR20291_1056	0	0	825	putative glycosyltransferase
CDR20291_1057	1	0.001039501	1068	conserved hypothetical protein
CDR20291_1071	0	0	672	hypothetical protein
CDR20291_1072	0	0	900	integrase
CDR20291_107A	0	0	414	hypothetical protein
CDR20291_1085	0	0	573	elongation factor P
CDR20291_1087	0	0	711	putative ribonuclease III
CDR20291_1090	0	0	1281	putative signal recognition particle
CDR20291_1091	0	0	369	putative DNA-binding regulator
CDR20291_1092	0	0	1344	signal recognition particle protein
CDR20291_1093	0	0	273	30S ribosomal protein S16
CDR20291_1095	0	0	516	putative 16S rRNA processing protein
CDR20291_1096	0	0	696	tRNA (Guanine-n(1)-)-methyltransferase

CDR20291_1097	0	0	351	50S ribosomal protein L19
CDR20291_1098	0	0	906	putative GTPase
CDR20291_1115	0	0	786	GTP-sensing transcriptional pleiotropic repressor
CDR20291_1119	0	0	1203	cysteine desulfurase
CDR20291_1120	0	0	441	NifU-like protein
CDR20291_1121	0	0	1080	putative tRNA (5-methylaminomethyl-2-thiouridylate)-methyltransferase
CDR20291_1122	0	0	2640	putative alanyl-tRNA synthetase
CDR20291_1124	0	0	2172	putative membrane protein
CDR20291_1130	0	0	201	putative small acid-soluble spore protein
CDR20291_1146	2	0.000516796	4299	DNA polymerase III PolC-type
CDR20291_1148	0	0	1155	transcription elongation protein
CDR20291_1149	0	0	279	uncharacterised protein
CDR20291_1150	0	0	312	putative ribosomal protein
CDR20291_1151	0	0	1941	translation initiation factor IF-2
CDR20291_1153	1	0.001098901	1011	putative RNA-binding protein
CDR20291_1155	0	0	930	riboflavin biosynthesis protein
CDR20291_1156	0	0	99	conserved hypothetical protein
CDR20291_1157	0	0	258	30S ribosomal protein S15
CDR20291_1159	1	0.000526039	2112	polyribonucleotide nucleotidyltransferase
CDR20291_1163	1	0.000907441	1224	aspartokinase
CDR20291_1168	0	0	552	CDP-diacylglycerol--glycerol-3-phosphate 3-phosphatidyltransferase
CDR20291_1170	0	0	1542	putative nucleic acid-binding protein
CDR20291_1171	2	0.001246883	1782	putative DNA helicase
CDR20291_1195	0	0	822	putative pyridoxine kinase
CDR20291_1197	0	0	201	putative cold shock protein
CDR20291_1202	0	0	321	putative phage repressor
CDR20291_1203	0	0	198	putative phage regulatory protein
CDR20291_1209	0	0	168	conserved hypothetical protein
CDR20291_1213	0	0	327	putative phage protein
CDR20291_1223	0	0	207	putative phage regulatory protein

CDR20291_1254	0	0	1449	GntR-family transcriptional regulator
CDR20291_1255	0	0	900	D-alanine--D-alanine ligase B
CDR20291_1298	0	0	573	putative GTP cyclohydrolase I
CDR20291_1299	1	0.001381215	804	dihydropteroate synthase
CDR20291_1300	0	0	366	dihydroneopterin aldolase
CDR20291_1301	0	0	507	2-amino-4-hydroxy-6-hydroxymethyldihydropteridi ne pyrophosphokinase
CDR20291_1303	0	0	1827	DNA primase
CDR20291_1304	0	0	1167	RNA polymerase sigma factor (sigma-43)
CDR20291_1338	1	0.000992063	1119	transposase-like protein b
CDR20291_1370	0	0	1209	tyrosyl-tRNA synthetase
CDR20291_1393	0	0	210	conserved hypothetical protein
CDR20291_1418B	0	0	342	Transcriptional regulator
CDR20291_1419A	0	0	144	hypothetical protein
CDR20291_1436A	0	0	234	putative phage protein
CDR20291_1459	0	0	183	hypothetical phage protein
CDR20291_1464A	0	0	177	hypothetical protein
CDR20291_1469	0	0	756	transposase-like protein b
CDR20291_1675	1	0.000966184	1149	putative methylase
CDR20291_1676	1	0.000799361	1389	putative lipoprotein
CDR20291_1677	0	0	735	two-component response regulator
CDR20291_1678	0	0	1434	two-component sensor histidine kinase
CDR20291_1712	0	0	597	putative acyltransferase
CDR20291_1713	1	0.001322751	840	putative 4-hydroxy-3-methylbut-2-enyl diphosphate reductase
CDR20291_1744	0	0	1881	site-specific recombinase
CDR20291_1745	0	0	351	hypothetical protein
CDR20291_1747	0	0	366	putative uncharacterized protein
CDR20291_1750	1	0.001157407	960	putative lantibiotic abc transporter
CDR20291_1752	1	0.001428571	777	putative lantibiotic abc transporter
CDR20291_1755	0	0	450	sigma-24 (feci)
CDR20291_1756	0	0	495	rna polymerase

CDR20291_1757	0	0	297	hypothetical protein
CDR20291_1758	0	0	1452	putative endonuclease relaxase Tn1549-like
CDR20291_1760	0	0	366	addiction module antitoxin
CDR20291_1762	0	0	735	phage protein
CDR20291_1767	0	0	480	hypothetical protein
CDR20291_1768	0	0	999	hypothetical protein
CDR20291_1769	0	0	558	hypothetical protein
CDR20291_1773	0	0	435	putative RNA polymerase sigma factor Tn5397
CDR20291_1778	0	0	432	hypothetical protein
CDR20291_1781	0	0	339	hypothetical protein
CDR20291_1782	0	0	294	hypothetical protein
CDR20291_1783	0	0	159	hypothetical protein
CDR20291_1784	0	0	1227	putative endonuclease relaxase Tn1549-like
CDR20291_1786	0	0	1329	hypothetical protein
CDR20291_1788	1	0.000664894	1671	hypothetical protein
CDR20291_1793	2	0.001240695	1791	putative DNA topoisomerase
CDR20291_1839	0	0	288	putative ethanolamine/propanediol utilization protein
CDR20291_1854	0	0	681	SOS regulatory protein
CDR20291_1859	0	0	945	acetyl-coenzyme A carboxylase carboxyl transferase subunit alpha
CDR20291_1860	0	0	855	acetyl-coenzyme A carboxylase carboxyl transferase subunit beta
CDR20291_1861	0	0	1362	biotin carboxylase (acetyl-CoA carboxylase subunit A)
CDR20291_1862	0	0	453	biotin carboxyl carrier protein of acetyl-CoA carboxylase
CDR20291_1906	1	0.000992063	1119	transposase-like protein b
CDR20291_1914A	0	0	183	hypothetical protein
CDR20291_1914B	0	0	177	conserved hypothetical protein
CDR20291_1920A	0	0	135	conserved hypothetical protein
CDR20291_1930	0	0	372	putative phage regulatory protein
CDR20291_1966	0	0	1665	putative glutaminyl-tRNA synthetase
CDR20291_2020	1	0.00086881	1278	two-component sensor histidine kinase
CDR20291_2029	1	0.00118624	936	putative cations transporter

CDR20291_2035	1	0.001051525	1056	4-hydroxy-3-methylbut-2-en-1-yl diphosphate synthase
CDR20291_2037	1	0.000961538	1155	1-deoxy-d-xylulose 5-phosphate reductoisomerase
CDR20291_2041	0	0	780	putative phosphatidate cytidylyltransferase
CDR20291_2042	0	0	735	undecaprenyl pyrophosphate synthetase
CDR20291_2044	0	0	558	ribosome recycling factor
CDR20291_2045	0	0	705	uridylate kinase
CDR20291_2046	0	0	912	elongation factor Ts
CDR20291_2047	0	0	714	30S ribosomal protein S2
CDR20291_2106	0	0	237	putative oxidoreductase
CDR20291_2144	0	0	1398	asparaginyl-tRNA synthetase
CDR20291_2193	0	0	186	hypothetical protein
CDR20291_2196	1	0.001356852	818	integrase
CDR20291_2197	0	0	672	transposase
CDR20291_2199	0	0	201	cold shock protein
CDR20291_2202	0	0	696	ABC transporter
CDR20291_2224	1	0.000740741	1500	inosine-5'-monophosphate dehydrogenase
CDR20291_2299A	0	0	318	hypothetical protein
CDR20291_2300	0	0	270	conserved hypothetical protein
CDR20291_2309	0	0	546	PTS system
CDR20291_2324	0	0	2067	glycyl-tRNA synthetase beta chain
CDR20291_2325	0	0	879	glycyl-tRNA synthetase alpha chain
CDR20291_2329	1	0.001242236	894	GTP-binding protein
CDR20291_2332	0	0	462	putative metal-dependent hydrolase
CDR20291_2339	0	0	180	30S ribosomal protein S21
CDR20291_2354	2	0.001201923	1848	chaperone protein
CDR20291_2355	0	0	621	heat shock protein
CDR20291_2366	0	0	267	30S ribosomal protein S20
CDR20291_2367	0	0	993	putative DNA polymerase III
CDR20291_2385	1	0.000413223	2688	putative sensor histidine kinase
CDR20291_2410	0	0	2421	leucyl-tRNA synthetase

CDR20291_2413	0	0	690	nicotinate-nucleotide adenylyltransferase
CDR20291_2443	0	0	498	phosphopantetheine adenylyltransferase
CDR20291_2460	0	0	642	putative thiamine pyrophosphokinase
CDR20291_2462	0	0	921	putative ATP/GTP-binding protein
CDR20291_2463	0	0	738	probable short chain dehydrogenase
CDR20291_2470	0	0	930	methionyl-tRNA formyltransferase
CDR20291_2472	0	0	2526	putative primosomal protein N'
CDR20291_2473	0	0	1242	coenzyme A biosynthesis bifunctional protein
CDR20291_2474	0	0	267	DNA-directed RNA polymerase omega chain
CDR20291_2475	0	0	618	guanylate kinase
CDR20291_2476	0	0	882	uncharacterised protein
CDR20291_2477	0	0	831	diaminopimelate epimerase
CDR20291_2493	0	0	1002	tryptophanyl-tRNA synthetase
CDR20291_2501	0	0	1119	transposase-like protein b
CDR20291_2502	0	0	3108	isoleucyl-tRNA synthetase
CDR20291_2506	0	0	459	Cell division protein SepF
CDR20291_2507	0	0	705	putative alanine racemase
CDR20291_2518	0	0	1020	glycerol-3-phosphate dehydrogenase [NAD(P)+]
CDR20291_2519	0	0	615	putative membrane protein
CDR20291_2526	0	0	693	two-component response regulator
CDR20291_2534	1	0.000956938	1161	cell division protein
CDR20291_2539	0	0	1227	UDP-N-acetylglucosamine--N-acetylmuramyl-(penta peptide) pyrophospho
CDR20291_2541	0	0	1356	UDP-N-acetylmuramoylalanine--D-glutamate ligase
CDR20291_2542	0	0	969	phospho-N-acetylmuramoyl-pentapeptide-transfera se
CDR20291_2543	0	0	1374	UDP-N-acetylmuramoyl-tripeptide--D-alanyl-D-ala nine ligase
CDR20291_2552	0	0	1455	putative UDP-N-acetylmuramoylalanyl-D-glutamate--2
CDR20291_2564	1	0.00094162	1179	putative acetyl-CoA acetyltransferase
CDR20291_2569	1	0.001197605	927	putative Calcium-chelating exported protein
CDR20291_2571	0	0	570	putative propanediol utilization protein
CDR20291_2602	1	0.00109529	1014	UDP-glucose 4-epimerase

CDR20291_2603	1	0.001175088	945	UTP--glucose-1-phosphate uridylyltransferase
CDR20291_2628	0	0	1788	putative aspartyl-tRNA synthetase
CDR20291_2629	0	0	1263	putative histidyl-tRNA synthetase
CDR20291_2630	0	0	1557	putative coproporphyrinogen III oxidase
CDR20291_2632	0	0	450	putative D-tyrosyl-tRNA protein
CDR20291_2633	2	0.001006036	2208	putative GTP pyrophosphokinase
CDR20291_2657	0	0	1914	capsular polysaccharide biosynthesis protein
CDR20291_2658	0	0	1092	putative capsular polysaccharide biosynthesis glycosyl transferase
CDR20291_2659	0	0	1344	putative UDP-glucose 6-dehydrogenase
CDR20291_2660	0	0	753	putative teichuronic acid biosynthesis glycosyl transferase
CDR20291_2661	0	0	909	putative beta-glycosyltransferase
CDR20291_2662	0	0	750	putative teichuronic acid biosynthesis glycosyl transferase
CDR20291_2663	1	0.000932836	1191	putative minor teichoic acid biosynthesis protein
CDR20291_2664	1	0.001322751	840	putative glycosyl transferase
CDR20291_2665	0	0	1158	putative polysaccharide polymerase
CDR20291_2666	0	0	723	putative polysaccharide biosynthesis protein
CDR20291_2668	1	0.000650618	1707	putative phosphomannomutase/phosphoglycerate mutase
CDR20291_2669	0	0	1560	putative transmembrane virulence factor MviN family protein
CDR20291_2670	0	0	1056	cell surface protein
CDR20291_2671	0	0	699	putative glycosyltransferase
CDR20291_2681	2	0.00094697	2346	preprotein translocase SecA subunit
CDR20291_2682	0	0	2277	cell surface protein (S-layer precursor protein)
CDR20291_2688A	0	0	144	conserved hypothetical protein
CDR20291_2694	1	0.001089325	1020	holliday junction DNA helicase
CDR20291_2709A	0	0	318	hypothetical protein
CDR20291_2723A	0	0	186	hypothetical protein
CDR20291_2834	0	0	1119	transposase-like protein b
CDR20291_2961B	0	0	138	hypothetical protein
CDR20291_3004	1	0.001317523	843	putative phage DNA-binding protein
CDR20291_3005	0	0	492	putative phage repressor

CDR20291_3006	0	0	771	putative phage protein
CDR20291_3011	0	0	456	SsrA-binding protein
CDR20291_3024	0	0	222	putative subunit of preprotein translocase
CDR20291_3026	1	0.000859107	1293	enolase
CDR20291_3027	0	0	1533	3-bisphosphoglycerate-independent phosphoglycerate mutase
CDR20291_3028	0	0	744	triosephosphate isomerase
CDR20291_3029	0	0	1203	phosphoglycerate kinase
CDR20291_3030	0	0	1008	glyceraldehyde-3-phosphate dehydrogenase 2
CDR20291_3081	0	0	1119	putative amidohydrolase
CDR20291_3084	0	0	1002	aspartate-semialdehyde dehydrogenase
CDR20291_3085	1	0.00125	888	dihydrodipicolinate synthase
CDR20291_3086	1	0.001481481	750	dihydrodipicolinate reductase
CDR20291_3087	0	0	717	5-tetrahydropyridine-2
CDR20291_3111	1	0.000854701	1299	putative folylpolyglutamate synthase
CDR20291_3114	1	0.000416493	2667	valyl-tRNA synthetase
CDR20291_3146	0	0	1350	glucose-6-phosphate isomerase
CDR20291_3161	0	0	585	probable GTP-binding protein
CDR20291_3162	1	0.000468823	2370	ATP-dependent protease La
CDR20291_3165	1	0.000888099	1251	ATP-dependent Clp protease ATP-binding subunit
CDR20291_3170	0	0	2034	DNA ligase
CDR20291_3187A	0	0	138	autoinducer prepeptide
CDR20291_3217	0	0	960	6-phosphofructokinase
CDR20291_3218	1	0.000311236	3570	DNA polymerase III alpha subunit
CDR20291_3219	1	0.00117096	948	putative sporulation transcription regulator whiA
CDR20291_3224	0	0	954	UDP-N-acetylenolpyruvoylglucosamine reductase
CDR20291_3261B	0	0	120	conserved hypothetical protein
CDR20291_3302	0	0	381	holo-[acyl-carrier protein] synthase
CDR20291_3303	0	0	261	ATP synthase epsilon chain (partial)
CDR20291_3304	0	0	1395	ATP synthase beta chain
CDR20291_3305	0	0	843	ATP synthase subunit gamma

CDR20291_3306	0	0	1503	ATP synthase alpha chain
CDR20291_3307	0	0	546	ATP synthase subunit delta
CDR20291_3308	0	0	516	ATP synthase B chain
CDR20291_3310	0	0	705	ATP synthase A chain
CDR20291_3311	0	0	387	ATP synthase protein I
CDR20291_3312	0	0	228	putative ATP synthase protein
CDR20291_3318	0	0	1062	putative RNA-binding protein
CDR20291_3320	0	0	1065	peptide chain release factor 1
CDR20291_3323	0	0	201	50S ribosomal protein L31
CDR20291_3333	0	0	279	DNA-binding protein HU
CDR20291_3335	0	0	1614	stage V sporulation protein B
CDR20291_3339	0	0	561	peptidyl-tRNA hydrolase
CDR20291_3351	0	0	951	ribose-phosphate pyrophosphokinase
CDR20291_3352	1	0.000805153	1380	bifunctional protein [includes: UDP-N- acetylglucosamine pyrophosphoryla
CDR20291_3355	0	0	1353	UDP-N-acetylmuramate--L-alanine ligase
CDR20291_3375	2	0.001146132	1938	methionyl-tRNA synthetase
CDR20291_3378	1	0.000776398	1431	putative nicotinate phosphribosyltransferase
CDR20291_3384	1	0.00118624	936	putative DNA polymerase III
CDR20291_3385	0	0	705	putative thymidylate kinase
CDR20291_3389	0	0	1530	lysyl-tRNA synthetase
CDR20291_3390	0	0	480	transcription elongation factor grea
CDR20291_3392	0	0	771	Type III pantothenate kinase (Pantothenic acid kinase) (PanK-III)
CDR20291_3397	0	0	1389	putative ATPase
CDR20291_3399	0	0	807	glutamate racemase
CDR20291_3403	0	0	921	4-diphosphocytidyl-2-c-methyl-D-erythritol kinase
CDR20291_3473A	0	0	141	hypothetical protein
CDR20291_3473B	0	0	192	hypothetical protein
CDR20291_3512	1	0.001048218	1059	DNA replication protein DnaD/DnaB
CDR20291_3513	0	0	993	putative DNA replication protein
CDR20291_3516	0	0	1329	replicative DNA helicase

CDR20291_3517	0	0	450	50S ribosomal protein L9
CDR20291_3518	1	0.000556793	1995	putative RNA/single-stranded DNA exonuclease
CDR20291_3519	1	0.001197605	927	putative membrane protein
CDR20291_3520	0	0	321	uncharacterised protein
CDR20291_3522	0	0	435	single-strand binding protein
CDR20291_3523	0	0	279	30S ribosomal protein S6
CDR20291_3525	0	0	186	conserved hypothetical protein
CDR20291_3526	0	0	264	conserved hypothetical protein
CDR20291_3535	2	0.001171646	1896	glucose inhibited division protein A
CDR20291_3540	0	0	345	ribonuclease P protein component
CDR20291_3541	0	0	135	50S ribosomal protein L34
CDR20291_3542	0	0	1320	chromosomal replication initiator protein
CDR20291_3543	0	0	1209	DNA polymerase III
CDR20291_3544	0	0	207	putative RNA-binding mediating protein
CDR20291_3545	0	0	1116	DNA replication and repair protein
CDR20291_3546	0	0	1902	DNA gyrase subunit B
CDR20291_3547	0	0	1664	DNA gyrase subunit A
