# Supplementary material for: High-Throughput Analysis of Gene Essentiality and Sporulation in Clostridium difficile
Source: mBio. 2015 Feb 24;6(2):e02383-14. doi: 10.1128/mBio.02383-14 (PMC4358009; doi:10.1128/mBio.02383-14)
Supplement: Table S2 — Genes required for sporulation in C. difficile R20291 [file mbo001152196st2.pdf]

locus_tag	function	logFC	q.value	630 orthologue locus_tag
CDR20291_0003	seryl-tRNA synthetase	-3.5327875	0.029669514	CD630_00140
CDR20291_0004	putative cytosine/adenosine deaminase	-10.776685	3.18E-09	CD630_00150
CDR20291_0006	DNA binding protein	-8.8436605	2.08E-05	CD630_00170
CDR20291_0007	recombination protein	-9.6375654	3.48E-06	CD630_00180
CDR20291_0018	putative membrane protein	-9.5763775	1.39E-06	CD630_00290
CDR20291_0020	AraC-family transcriptional regulator	-2.8246041	1.31E-14	CD630_00310
CDR20291_0025	acetoin:2,6-dichlorophenolindophenol oxidoreductase alpha subunit	-7.552354	4.01E-13	CD630_00360
CDR20291_0027	E2 component of acetoin dehydrogenase enzyme system (dihydrolipoamide acetyltransferase)	-2.3049538	1.17E-07	CD630_00380
CDR20291_0028	E3 component of acetoin dehydrogenase enzyme system (dihydrolipoyl dehydrogenase)	-2.1159482	4.80E-15	CD630_00390
CDR20291_0039	putative dual-specificity prolyl/cysteinyl-tRNA synthetase	-2.6848693	1.38E-10	CD630_00500
CDR20291_0050	RNA polymerase sigma-H factor	-9.8902176	1.73E-39	CD630_00570
CDR20291_0051	elongation factor TU	-9.4206022	2.42E-06	
CDR20291_0105	aspartate aminotransferase	-7.6854612	1.10E-06	CD630_01070
CDR20291_0123	stage II sporulation protein D	-8.9889938	1.70E-43	CD630_01240
CDR20291_0124	putative cell wall endopeptidase	-6.6661979	2.34E-14	CD630_01250
CDR20291_0125	stage III sporulation protein D	-10.122604	3.49E-07	CD630_01260
CDR20291_0128	putative sporulation protein yyac	-3.8524059	1.95E-06	CD630_01290
CDR20291_0130	putative membrane protein	-12.533059	3.32E-19	CD630_01310
CDR20291_0138	glycosylasparaginase	-2.0396298	9.27E-06	CD630_01390
CDR20291_0139	putative peptidase	-2.3220736	5.07E-13	CD630_01400
CDR20291_0140	putative copper homeostasis protein	-3.0088435	0.002241391	CD630_01410
CDR20291_0160	Radical SAM-family protein	-2.5008949	4.17E-07	CD630_01620
CDR20291_0162	conserved hypothetical protein	-9.27897	6.74E-06	CD630_01631
CDR20291_0163	uncharacterised protein	-3.8878336	0.019823432	CD630_01632
CDR20291_0167	putative sigma-54-dependent transcriptional regulator	-2.3171071	3.40E-19	CD630_01670
CDR20291_0171	putative redox-sensing transcriptional repressor	-11.612673	6.33E-12	CD630_01710
CDR20291_0180	NAD-specific glutamate dehydrogenase	-5.5543167	0.000141963	CD630_01790
CDR20291_0202	putative chromate transporter	-4.4774238	0.007083376	CD630_02010
CDR20291_0203	hypothetical protein	-3.2413306	8.27E-21	
CDR20291_0207	PTS system, IIa component	-10.987475	1.68E-09	CD630_02060
CDR20291_0209	PTS system, IIb component	-4.8681921	3.40E-10	CD630_02080
CDR20291_0210	putative sugar-phosphate kinase	-4.6346951	1.11E-06	CD630_02090
CDR20291_0213	uncharacterised protein	-5.1695559	0.000569921	CD630_02140
CDR20291_0225	dtdp-4-dehydrorhamnose 3,5-epimerase	-3.5262787	3.10E-25	
CDR20291_0235	carbon storage regulator	-3.8064794	0.019541895	CD630_02340
CDR20291_0238	flagellar cap protein	-3.4598442	4.70E-20	CD630_02370

CDR20291_0250	flagellar hook-basal body complex protein	-4.18161	9.51E-07	CD630_02470
CDR20291_0255	flagellar protein	-2.0920942	0.000507373	CD630_02520
CDR20291_0265	flagellar export protein	-10.707733	1.15E-08	CD630_02610
CDR20291_0275	putative flagellar motor switch protein	-3.7441718	9.82E-09	CD630_02710
CDR20291_0280	AraC-family transcriptional regulator	-2.0325985	0.001720723	CD630_02760
CDR20291_0281	conserved hypothetical protein	-3.3154232	0.000445708	CD630_02770
CDR20291_0287	PTS system, IIa component	-3.965708	0.000981009	CD630_02840
CDR20291_0288	PTS system, IIb component	-3.961595	8.89E-05	CD630_02850
CDR20291_0289	putative PTS system, IIa component	-11.213343	1.62E-09	CD630_02860
CDR20291_0293	conserved hypothetical protein	-9.5009691	3.37E-06	CD630_02900
CDR20291_0294	putative peptidase	-3.8543431	7.18E-35	CD630_02910
CDR20291_0296	ABC transporter, ATP-binding protein	-2.5181185	0.001114619	CD630_02930
CDR20291_0304	ribose ABC transporter, ATP-binding protein	-2.8925491	4.39E-15	CD630_03010
CDR20291_0316	putative spore coat assembly asparagine-rich protein	-2.4605236	0.001066619	CD630_03110
CDR20291_0317	ArsR-family transcriptional regulator	-4.2447212	1.91E-08	CD630_03120
CDR20291_0318	putative heavy-metal-transporting ATPase	-8.6173176	4.78E-45	CD630_03130
CDR20291_0339	aldehyde-alcohol dehydrogenase [includes: alcohol dehydrogenase; acetaldehyde dehydrogenase [acetyl	-2.245568	6.87E-08	CD630_03340
CDR20291_0340	conserved hypothetical protein	-4.2165255	0.005322813	CD630_03350
CDR20291_0343	two-component sensor histidine kinase	-3.3826227	1.27E-13	CD630_03380
CDR20291_0354	putative hydrolase, HAD superfamily	-5.8987366	5.42E-06	CD630_03500
CDR20291_0355	conserved hypothetical protein	-5.3140584	4.09E-51	CD630_03510
CDR20291_0358	putative membrane protein	-3.9678825	1.21E-09	CD630_03541
CDR20291_0360	6-phospho-beta-glucosidase	-3.4523199	1.72E-13	CD630_03890
CDR20291_0361	beta-glucoside bgl operon transcription antiterminator	-2.7399926	0.004371175	CD630_03900
CDR20291_0366	isocaprenoyl-CoA:2-hydroxyisocaproate CoA-transferase	-10.894377	1.84E-09	CD630_03950
CDR20291_0367	activator of 2-hydroxyisocaproyl-CoA dehydratase	-9.4585771	3.58E-06	CD630_03960
CDR20291_0370	acyl-CoA dehydrogenase, short-chain specific	-7.8376217	2.96E-06	CD630_03990
CDR20291_0372	electron transfer flavoprotein alpha-subunit	-9.4258895	2.21E-06	CD630_04010
CDR20291_0384	putative arginine utilization sigma-54 dependent regulatory protein	-2.0536671	2.72E-07	CD630_04410
CDR20291_0385	putative oxidoreductase	-2.3044917	1.06E-08	CD630_04420
CDR20291_0395	AraC-family transcriptional regulator	-2.0349127	3.06E-16	CD630_04540
CDR20291_0400	ABC transporter, ATP-binding protein	-3.8287439	1.12E-10	CD630_04590
CDR20291_0401	ABC transporter, permease protein	-2.9638898	3.09E-25	CD630_04600
CDR20291_0409	putative hydrolase	-3.4001296	3.52E-05	CD630_04670
CDR20291_0418	conserved hypothetical protein	-2.3758893	1.52E-06	CD630_04760
CDR20291_0420	putative lantibiotic ABC transporter, ATP-binding protein	-11.517562	1.07E-11	CD630_04780
CDR20291_0427	hypothetical protein	-2.6739267	9.58E-08	
CDR20291_0429	two-component response regulator	-2.3497478	0.005517726	CD630_04860
CDR20291_0431	quaternary ammonium compound-resistance protein	-2.4582461	0.036754988	CD630_04880

CDR20291_0433	putative sugar-phosphate dehydrogenase	-11.877644	7.12E-12	CD630_04900
CDR20291_0434	PTS system, IIa component	-3.9786924	0.014223699	CD630_04910
CDR20291_0436	PTS system, IIc component	-2.3706814	7.33E-07	CD630_04930
CDR20291_0440	cell surface protein (putative hemagglutinin/adhesin)	-2.0011262	1.97E-09	CD630_05140
CDR20291_0456	DeoR-family transcriptional regulator	-4.8211627	2.77E-16	CD630_05310
CDR20291_0475	putative membrane protein	-3.6525659	0.014081308	CD630_05500
CDR20291_0479	signal peptidase I	-6.9455614	1.08E-46	CD630_05540
CDR20291_0480	singal peptidase I	-10.244453	3.95E-104	CD630_05550
CDR20291_0481	putative endonuclease	-8.275233	0.000105538	CD630_05560
CDR20291_0485	endonuclease IV	-2.9105184	0.001161197	CD630_05600
CDR20291_0496	putative sporulation protein	-3.3829355	8.76E-19	CD630_05720
CDR20291_0503	two-component sensor histidine kinase	-4.3825355	1.87E-62	CD630_05760
CDR20291_0505	putative efflux protein	-2.3747999	1.93E-16	CD630_05780
CDR20291_0507	NADP-dependent glyceraldehyde-3-phosphate dehydrogenase	-4.5197454	5.95E-19	CD630_05800
CDR20291_0510	putative signaling protein	-11.440107	3.46E-11	
CDR20291_0511	uncharacterised protein	-10.384372	4.34E-07	CD630_05870
CDR20291_0512	uncharacterised protein	-3.2730145	0.004463218	CD630_05880
CDR20291_0518	conserved hypothetical protein	-2.4705221	0.007557645	CD630_05930
CDR20291_0524	methylated-DNA--protein-cysteine methyltransferase 1	-9.8522209	3.10E-07	CD630_06010
CDR20291_0535	putative ABC transporter, permease protein	-2.4530513	2.33E-22	CD630_06120
CDR20291_0536	ABC transporter, ATP-binding protein	-10.81273	3.49E-09	CD630_06130
CDR20291_0545	putative phage-related regulatory protein	-5.4406232	2.60E-06	CD630_06240
CDR20291_0547	putative phage-related replicative helicase	-2.4066459	3.41E-07	CD630_06260
CDR20291_0548	putative phage positive regulator of late transcription	-9.6475885	1.66E-21	CD630_06270
CDR20291_0549	putative ferredoxin	-9.2579602	1.06E-10	CD630_06271
CDR20291_0550	putative membrane protein	-4.4323737	0.004099397	CD630_06280
CDR20291_0556	conserved hypothetical protein	-2.23484	5.40E-05	CD630_06340
CDR20291_0558	putative radical SAM superfamily protein	-2.024176	1.69E-16	CD630_06360
CDR20291_0576	putative ABC transporter, permease protein	-3.967604	1.43E-29	CD630_06540
CDR20291_0579	putative Na(+)/H(+) antiporter	-5.8869075	4.13E-05	CD630_06570
CDR20291_0586	conserved hypothetical protein	-2.2779288	0.001945593	CD630_06641
CDR20291_0588	putative lantibiotic ABC transporter, permease protein	-2.8785248	0.000523814	CD630_06650
CDR20291_0590	putative lantibiotic ABC transporter, ATP-binding protein	-11.348554	1.62E-10	CD630_06670
CDR20291_0598	putative acetyltransferase	-4.2285491	0.003465589	CD630_06750
CDR20291_0599	Extracytoplasmic function (ECF) anti-sigma factor rsiT	-2.1100736	2.43E-05	CD630_06760
CDR20291_0601	putative membrane protein	-2.0592677	0.000706411	
CDR20291_0604	hypothetical protein	-2.9032562	1.89E-10	
CDR20291_0621	putative exported peptidase	-2.3840707	1.49E-24	CD630_06950
CDR20291_0635	putative DNA mismatch repair protein	-2.9337787	4.66E-11	CD630_07090

CDR20291_0640	tetratricopeptide repeat protein	-4.0058679	4.40E-40	CD630_07140
CDR20291_0644	putative carbon monoxide dehydrogenase accessory protein	-4.9518879	3.82E-34	CD630_07170
CDR20291_0648	uncharacterised protein	-3.7408926	7.21E-09	CD630_07210
CDR20291_0654	putative carbon monoxide dehydrogenase/acetyl-CoA synthase complex, methyltransferase subunit	-2.5357641	0.005104783	CD630_07270
CDR20291_0659	putative radical SAM superfamily protein	-4.8643122	6.31E-34	CD630_07320
CDR20291_0670	putative ethanolamine transporter	-10.444163	1.13E-07	CD630_07420
CDR20291_0688	Ca2+/Na+ antiporter	-6.2789128	1.01E-18	CD630_07600
CDR20291_0691	glucitol operon activator protein	-3.4936973	0.000753796	CD630_07630
CDR20291_0693	PTS system, glucitol/sorbitol-specific IIb component	-3.0513559	7.06E-05	CD630_07650
CDR20291_0695	PTS system, glucitol/sorbitol-specific IIa component	-12.340831	8.05E-17	CD630_07670
CDR20291_0696	sorbitol-6-phosphate 2-dehydrogenase	-10.267893	8.27E-07	CD630_07680
CDR20291_0699	anti-sigma F factor antagonist	-10.91624	2.72E-09	CD630_07700
CDR20291_0700	anti-sigma F factor	-9.8278862	1.54E-06	CD630_07710
CDR20291_0701	RNA polymerase sigma-F factor	-5.7497318	5.00E-06	CD630_07720
CDR20291_0702	stage V sporulation protein AC	-11.011033	1.61E-09	CD630_07730
CDR20291_0703	stage V sporulation protein AD	-6.1919712	4.58E-08	CD630_07740
CDR20291_0704	stage V sporulation protein AE	-14.172243	1.16E-73	CD630_07750
CDR20291_0707	putative membrane protein	-2.9351842	0.000318359	CD630_07780
CDR20291_0713	putative exported protein	-5.4153374	1.17E-21	CD630_07820
CDR20291_0714	putative stage IV sporulation protein	-7.2816857	5.23E-18	CD630_07830
CDR20291_0723	putative membrane protein	-8.6911581	9.57E-09	CD630_07920
CDR20291_0724	putative membrane protein	-10.568752	3.65E-07	CD630_07930
CDR20291_0727	LacI-family transcriptional regulator	-5.6292992	1.12E-05	CD630_07960
CDR20291_0730	putative beta-alanine CoA-transferase	-11.689106	1.79E-11	CD630_07990
CDR20291_0732	probable permease	-10.575861	1.89E-08	CD630_08010
CDR20291_0737	putative sigma-54-dependent transcriptional regulator	-2.7232649	8.74E-14	CD630_08060
CDR20291_0740	conserved hypothetical protein	-4.4229866	2.67E-15	CD630_08090
CDR20291_0746	probable transporter	-3.1079811	1.05E-36	CD630_08150
CDR20291_0748	putative 6-phospho-beta-glucosidase	-8.3830244	1.17E-16	CD630_08180
CDR20291_0751	two-component sensor histidine kinase	-3.8148414	0.000376202	CD630_08210
CDR20291_0753	putative lantibiotic ABC transporter, permease protein	-9.9365727	2.34E-07	CD630_08230
CDR20291_0755	rubrerythrin	-2.5824364	2.98E-05	CD630_08250
CDR20291_0762	putative homocitrate/2-isopropylmalate synthase	-4.4329255	9.64E-43	CD630_08320
CDR20291_0763	putative aconitase/3-isopropylmalate dehydratase	-5.8525878	1.52E-19	CD630_08330
CDR20291_0764	putative isocitrate/3-isopropylmalate dehydrogenase	-4.8619345	1.31E-16	CD630_08340
CDR20291_0775	putative nuclease	-2.599153	0.005298735	CD630_08450
CDR20291_0777	conserved hypothetical protein	-2.8564319	0.000224539	CD630_08470
CDR20291_0778	conserved hypothetical protein	-2.7445102	1.43E-11	CD630_08480
CDR20291_0781	putative membrane protein	-2.2539225	5.16E-06	CD630_08510

CDR20291_0789	conserved hypothetical protein	-11.417522	2.58E-11	CD630_08600
CDR20291_0790	PTS system, IIb component	-12.77138	1.85E-24	CD630_08610
CDR20291_0803	ABC transporter, ATP-binding protein	-3.2220371	0.006482613	CD630_08740
CDR20291_0804	ABC transporter, permease protein	-4.0409232	0.011720686	CD630_08750
CDR20291_0806	ABC transporter, ATP-binding protein	-5.6375353	7.76E-05	CD630_08770
CDR20291_0814	glycogen synthase	-2.7624165	1.23E-10	CD630_08840
CDR20291_0832	putative cation efflux rotein	-2.9929115	0.002211243	CD630_09020
CDR20291_0839	putative 30S ribosomal protein S1	-2.7735411	8.22E-08	CD630_09860
CDR20291_0854	ABC transporter, ATP-binding protein	-2.0793452	0.007062989	CD630_10010
CDR20291_0855	putative regulatory protein	-2.8886507	2.96E-06	CD630_10020
CDR20291_0859	conserved hypothetical protein	-10.32878	2.48E-07	CD630_10070
CDR20291_0866	N-acetylglucosamine-6-phosphate deacetylase	-7.3565468	4.95E-08	CD630_10100
CDR20291_0867	glucosamine-6-phosphate deaminase	-13.459828	3.43E-42	CD630_10110
CDR20291_0879	putative transcriptional regulator	-2.8854237	1.17E-08	CD630_10230
CDR20291_0880	spermidine/putrescine ABC transporter, ATP-binding protein	-6.4304472	3.93E-56	CD630_10240
CDR20291_0881	spermidine/putrescine ABC transporter, permease protein	-6.3968073	1.48E-34	CD630_10250
CDR20291_0882	putative spermidine/putrescine ABC transporter, permease protein	-3.4842322	2.74E-05	CD630_10260
CDR20291_0883	spermidine/putrescine ABC transporter, substrate-binding lipoprotein	-11.713274	4.29E-62	CD630_10270
CDR20291_0895	conserved hypothetical protein	-5.5611369	4.89E-19	CD630_10390
CDR20291_0896	ATP-dependent nuclease subunit B	-10.848096	1.05E-08	CD630_10400
CDR20291_0897	ATP-dependent nuclease subunit A	-5.3153441	0.000545585	CD630_10410
CDR20291_0898	nuclease subunit D	-2.009233	1.20E-10	CD630_10420
CDR20291_0918	acyl carrier protein	-14.373005	3.28E-82	CD630_10620
CDR20291_0920	uncharacterised protein	-12.258843	1.13E-15	CD630_10631
CDR20291_0922	conserved hypothetical protein	-9.3096318	4.06E-06	CD630_10633
CDR20291_0923	LacI-family transcriptional regulator (catabolite control protein)	-12.496422	5.50E-173	CD630_10640
CDR20291_0924	uncharacterised protein	-9.8139025	7.80E-06	CD630_10650
CDR20291_0926	uncharacterised protein	-3.0771877	5.10E-07	CD630_10670
CDR20291_0927	putative polysaccharide biosynthesis/sporulation protein	-2.7564596	4.16E-14	CD630_10680
CDR20291_0930	uncharacterised protein, UPF0291 protein family	-3.3788815	5.93E-05	CD630_10710
CDR20291_0935	PTS system, IIb component	-4.2486006	0.001229029	CD630_10760
CDR20291_0936	PTS system, IIc component	-2.37709	0.021855143	CD630_10770
CDR20291_0938	LysR-family transcriptional regulator	-4.9331953	0.00292519	CD630_10790
CDR20291_0948	two-component response regulator	-2.7871159	4.38E-09	CD630_10890
CDR20291_0950	putative lipoprotein	-9.3332255	8.27E-06	CD630_11190
CDR20291_0964	DNA polymerase I	-3.3857694	9.22E-11	CD630_11280
CDR20291_0966	putative transglycosylase	-10.585413	1.24E-08	CD630_11300
CDR20291_0972	putative membrane protein	-3.5425097	6.87E-22	CD630_11360
CDR20291_0973	electron transport complex protein	-11.442678	5.76E-11	CD630_11370

CDR20291_0974	electron transport complex protein	-10.13791	1.19E-07	CD630_11380
CDR20291_0975	electron transport complex protein	-8.8654105	2.19E-05	CD630_11390
CDR20291_0976	electron transport complex protein	-10.629614	8.14E-09	CD630_11400
CDR20291_0977	electron transport complex protein	-9.4717932	1.93E-06	CD630_11410
CDR20291_0978	electron transport complex protein	-2.5560561	0.026519833	CD630_11420
CDR20291_0997	ribonuclease g	-4.5765823	2.21E-22	CD630_11600
CDR20291_1009	electron transfer flavoprotein alpha-subunit	-2.3348615	2.16E-07	CD630_11720
CDR20291_1011	conserved hypothetical protein, DUF795 family,UPF0348 family	-2.9237025	4.53E-19	CD630_11740
CDR20291_1012	acetate kinase	-3.7688823	0.022740337	CD630_11750
CDR20291_1013	conserved hypothetical protein, DUF177 family	-10.602775	2.03E-06	CD630_11760
CDR20291_1028	putative acetyltransferase	-3.5492151	0.033976282	CD630_11900
CDR20291_1029	putative fructose-1,6-bisphosphatase	-4.2417183	0.010568657	CD630_11910
CDR20291_1030	stage III sporulation protein AA	-12.545707	6.93E-20	CD630_11920
CDR20291_1031	stage III sporulation protein AB	-10.928114	1.59E-09	CD630_11930
CDR20291_1033	stage III sporulation protein AD	-10.059333	1.41E-07	CD630_11950
CDR20291_1034	stage III sporulation-related protein	-13.133801	5.73E-34	CD630_11960
CDR20291_1035	putative stage III sporulation protein AF	-5.4877456	0.00025202	CD630_11970
CDR20291_1036	stage iii sporulation protein ag	-10.126936	5.31E-06	CD630_11980
CDR20291_1042	exodeoxyribonuclease VII small subunit	-9.5476272	4.21E-06	CD630_12040
CDR20291_1043	geranyltranstransferase	-11.0791	1.06E-08	CD630_12050
CDR20291_1045	1-deoxy-D-xylulose 5-phosphate synthase	-5.1678553	0.001156434	CD630_12070
CDR20291_1046	putative RNA methyltransferase	-2.4974994	0.012279149	CD630_12080
CDR20291_1051	stage IV sporulation protein B	-4.0728792	1.47E-10	CD630_12130
CDR20291_1052	stage 0 sporulation protein A	-10.527673	7.60E-23	CD630_12140
CDR20291_1067	putative penicillin-binding protein	-4.7764618	2.75E-35	CD630_12290
CDR20291_1067A	hypothetical protein	-3.9620026	5.97E-05	
CDR20291_1068	site-specific recombinase	-3.7990802	3.72E-17	CD630_12310
CDR20291_1072A	putative phage regulator	-4.4199472	0.003857597	CD630_12333
CDR20291_1073	putative phage protein	-4.4480779	0.005837398	CD630_12340
CDR20291_1075	putative exported protein	-4.5361861	0.004449344	
CDR20291_1080	putative phage-related DNA-directed RNA polymerase 7 kDa polypeptide	-8.9614809	1.24E-05	CD630_12401
CDR20291_1080A	hypothetical protein	-6.0625102	1.43E-05	
CDR20291_1089	chromosome partition protein	-3.5088822	0.000165635	CD630_12500
CDR20291_1099	branched-chain amino acid transport system carrier protein	-9.6166083	1.06E-11	CD630_12590
CDR20291_1114	DNA topoisomerase I	-5.6042468	0.00011199	CD630_12740
CDR20291_1118	RRF2-family transcriptional regulator	-9.6321584	2.09E-06	CD630_12780
CDR20291_1125	putative holliday junction resolvase	-8.4085545	0.000148383	CD630_12850
CDR20291_1127	ferric uptake regulation protein	-10.65438	9.36E-08	CD630_12870
CDR20291_1129	metallo beta-lactamase superfamily protein	-10.733695	7.33E-09	CD630_12890

CDR20291_1131	D-alanyl-D-alanine carboxypeptidase (penicillin-binding protein)	-5.2012633	1.26E-06	CD630_12910
CDR20291_1133	AraC-family transcriptional regulator	-11.151509	3.93E-10	CD630_12930
CDR20291_1135	segregation and condensation protein A	-4.8114308	0.000484848	CD630_12950
CDR20291_1136	segregation and condensation protein B	-5.1590839	9.99E-10	CD630_12960
CDR20291_1138	putative sporulation protein	-6.0828947	2.23E-05	CD630_12980
CDR20291_1141	putative membrane protein	-2.7021621	2.99E-19	CD630_13010
CDR20291_1142	conserved hypothetical protein	-9.7711616	5.09E-07	CD630_13011
CDR20291_1152	ribosome-binding factor A	-10.143319	1.87E-07	CD630_13100
CDR20291_1160	putative polysaccharide deacetylase	-4.3418416	0.001730979	CD630_13190
CDR20291_1161	putative peptidase	-6.1985275	3.94E-08	CD630_13200
CDR20291_1162	putative sporulation protein	-9.0138663	1.67E-05	CD630_13210
CDR20291_1165	putative DNA translocase	-7.4809908	2.18E-16	CD630_13240
CDR20291_1166	putative ATP/GTP binding protein	-10.994947	1.22E-08	CD630_13250
CDR20291_1167	radical SAM-superfamily protein	-11.174811	1.36E-08	CD630_13260
CDR20291_1169	RecA protein (recombinase A)	-9.9164617	2.49E-07	CD630_13280
CDR20291_1171	putative DNA helicase, UvrD/REP type	-8.685407	2.59E-05	CD630_13300
CDR20291_1173	undecaprenyl-diphosphatase	-4.6672712	0.000589956	CD630_13320
CDR20291_1185	glutamine synthetase	-2.3047343	8.93E-20	CD630_13430
CDR20291_1191	putative lantibiotic ABC transporter, ATP-binding protein	-2.8599954	5.11E-07	CD630_13490
CDR20291_1192	putative lantibiotic ABC transporter, permease protein	-3.7262661	0.002005453	CD630_13500
CDR20291_1201	putative phage-related protein	-2.1208906	0.000943335	CD630_13590
CDR20291_1212	putative phage cell wall hydrolase	-3.1970518	0.00048663	CD630_13680
CDR20291_1219	putative phage-related protein	-2.4684269	0.002704431	CD630_13750
CDR20291_1224	conserved hypothetical protein	-2.5384784	0.015222514	CD630_13782
CDR20291_1230	putative lamB-like protein, UPF0271 family	-2.6023898	4.81E-11	CD630_13840
CDR20291_1233	putative allophanate hydrolase subunit 2	-2.9179488	2.56E-12	CD630_13870
CDR20291_1235	putative chloromuconate cycloisomerase	-3.1429804	0.001522738	CD630_13890
CDR20291_1236	putative membrane protein	-4.2076622	3.96E-07	CD630_13900
CDR20291_1238	putative endoribonuclease	-8.8129506	2.05E-05	CD630_13920
CDR20291_1239	putative membrane protein	-2.0847165	2.21E-10	CD630_13930
CDR20291_1260	putative membrane protein	-6.8933878	3.83E-07	CD630_14130
CDR20291_1263	putative membrane protein	-10.539933	6.14E-08	CD630_14160
CDR20291_1265A	hypothetical protein	-10.215038	9.39E-08	
CDR20291_1271	conserved hypothetical protein	-3.7826015	0.020236304	CD630_14240
CDR20291_1274	putative transcriptional regulator	-2.2292868	1.51E-10	CD630_14280
CDR20291_1275	conserved hypothetical protein	-9.6150023	1.09E-06	CD630_14281
CDR20291_1276	putative acyl-CoA N-acyltransferase	-4.5966572	0.000225005	CD630_14290
CDR20291_1281	putative ribonuclease	-4.8519151	0.001436812	CD630_14320
CDR20291_1297	conserved hypothetical protein	-7.8020893	3.54E-11	CD630_14480

CDR20291_1305	putative SAM-dependent methyltransferase	-3.3898367	0.003791221	CD630_14560
CDR20291_1312	putative membrane protein	-10.341952	5.10E-08	CD630_14630
CDR20291_1314	two-component sensor kinase	-2.0619394	4.93E-06	CD630_14650
CDR20291_1319	putative phage shock protein	-5.0064385	0.001229068	CD630_14700
CDR20291_1328	ferrous iron transport protein B	-2.4764176	3.34E-11	CD630_14790
CDR20291_1332	putative aliphatic sulfonates ABC transporter, ATP-binding protein	-2.5936537	0.009474012	CD630_14830
CDR20291_1336	conserved hypothetical protein	-5.5109562	0.000168746	CD630_14870
CDR20291_1352	ABC transporter, ATP-binding protein	-10.652173	7.04E-09	CD630_15040
CDR20291_1368	putative acetyltransferase	-9.8037479	4.06E-07	CD630_15190
CDR20291_1373	putative rubrerythrin	-13.376506	1.90E-40	CD630_15240
CDR20291_1378	putative ABC transporter, permease protein	-3.6555434	8.93E-06	CD630_15290
CDR20291_1385	putative dehydrogenase, electron transfer subunit	-10.785405	4.72E-09	CD630_15360
CDR20291_1386	putative glutamate synthase [NADPH] small chain	-9.0529749	9.28E-10	CD630_15370
CDR20291_1395	putative hemolysin-like membrane protein	-2.0905115	0.000798475	CD630_15460
CDR20291_1396	putative ATP phosphoribosyltransferase regulatory subunit	-3.5929571	0.000106005	CD630_15470
CDR20291_1399	imidazoleglycerol-phosphate dehydratase	-4.0958001	1.20E-06	CD630_15500
CDR20291_1401	putative 1-(5-phosphoribosyl)-5-[(5-phosphoribosylamino)methyliden eamino] imidazole-4-carboxamide	-2.3890195	2.02E-07	CD630_15520
CDR20291_1402	putative imidazole glycerol phosphate synthase subunit	-4.2302665	0.00948113	CD630_15530
CDR20291_1412	conserved hypothetical protein	-9.5822898	1.11E-06	CD630_15640
CDR20291_1415	prophage lambdaba04, site-specific recombinase, phage integrase family	-4.0929487	7.76E-15	
CDR20291_1418C	hypothetical protein	-11.105842	1.57E-09	
CDR20291_1421A	hypothetical protein	-11.649698	3.52E-11	
CDR20291_1422	hypothetical protein	-11.873125	7.37E-13	
CDR20291_1423	hypothetical protein	-5.9236885	3.48E-05	
CDR20291_1424	dna-directed dna polymerase i	-11.841823	2.57E-12	
CDR20291_1425	putative virulence-associated protein e	-4.6430889	1.88E-24	
CDR20291_1427	hypothetical protein	-4.0505433	2.24E-05	
CDR20291_1429	hypothetical protein	-2.4112183	5.90E-16	
CDR20291_1433	phage portal protein	-6.1337501	1.05E-05	CD630_29210
CDR20291_1434A	hypothetical protein	-10.76389	3.30E-09	
CDR20291_1437	hypothetical phage protein	-11.680054	4.57E-12	CD630_09500
CDR20291_1438	phage protein	-10.372478	1.39E-07	CD630_09510
CDR20291_1441	phage protein	-11.695714	5.91E-12	CD630_29100
CDR20291_1442	phage protein	-9.9638981	3.96E-07	CD630_09550
CDR20291_1444	phage protein	-11.340134	8.21E-11	CD630_09560
CDR20291_1452	phage protein	-8.8173502	3.61E-05	CD630_09620
CDR20291_1453	phage protein	-8.6472871	5.96E-05	CD630_29010
CDR20291_1456	putative phage tail fiber protein	-10.696885	4.90E-08	CD630_09660
CDR20291_1463	n-acetylmuramoyl-l-alanine amidase (cell wall hydrolase)	-2.6541347	0.003852484	

CDR20291_1475	putative membrane-associated ribonuclease	-2.3279359	9.54E-05	CD630_15780
CDR20291_1478	uncharacterised protein	-6.7815721	6.85E-05	CD630_15810
CDR20291_1480	conserved hypothetical protein	-2.7892519	0.003155554	CD630_15830
CDR20291_1487	putative ribose ABC transporter, substrate-binding lipoprotein	-5.2613524	0.000190625	CD630_15890
CDR20291_1491	potassium-transporting ATPase C chain	-4.6448953	0.003149919	CD630_15930
CDR20291_1492	putative O-acetylserine sulfhydrylase	-10.030648	2.16E-14	CD630_15940
CDR20291_1493	serine acetyltransferase	-9.8855702	8.66E-07	CD630_15950
CDR20291_1496	putative endonuclease	-2.7132229	8.96E-06	CD630_15980
CDR20291_1497	putative phosphomethylpyrimidine kinase	-9.4673827	1.62E-10	CD630_15990
CDR20291_1502	ABC transporter, ATP-binding protein	-9.5993257	1.09E-31	CD630_16040
CDR20291_1503	putative transcriptional regulator	-8.5129046	4.59E-05	CD630_16050
CDR20291_1509	putative exported protein	-2.4173316	4.53E-12	CD630_16110
CDR20291_1524	putative D-alanine:D-alanine ligase (putative vancomycin/teicoplanin A-type resistance protein)	-10.188924	1.37E-07	CD630_16260
CDR20291_1529	putative superoxide dismutase [Mn]	-6.7712534	3.45E-07	CD630_16310
CDR20291_1545	putative iron compound ABC transporter, permease protein	-12.134901	3.55E-14	CD630_16470
CDR20291_1552	putative lipoate-protein ligase	-3.4271595	5.16E-05	CD630_16540
CDR20291_1558	uncharacterised protein	-5.0189713	0.00094661	CD630_16600
CDR20291_1560A	hypothetical protein	-3.5364399	0.042835382	CD630_16632
CDR20291_1565	putative membrane protein	-3.9508973	5.43E-12	CD630_16680
CDR20291_1569	two-component sensor histidine kinase	-2.0295037	1.67E-10	CD630_16720
CDR20291_1575	putative membrane protein	-10.356629	5.95E-08	CD630_16780
CDR20291_1576	conserved hypothetical protein	-10.42753	2.83E-08	CD630_16781
CDR20291_1578	putative FMN-binding exported protein	-2.6224225	1.21E-06	CD630_16800
CDR20291_1586	two-component response regulator	-4.3177221	5.06E-22	CD630_16880
CDR20291_1606	putative C4-dicarboxylate anaerobic carrier	-3.3168302	0.009314469	CD630_17070
CDR20291_1615	probable permease	-2.9472889	0.001829534	CD630_17160
CDR20291_1621	conserved hypothetical protein	-2.7069794	0.013995762	CD630_17240
CDR20291_1622	AraC-family transcriptional regulator	-2.2587423	2.41E-06	CD630_17250
CDR20291_1626A	conserved hypothetical protein	-2.0820686	0.013777316	CD630_17292
CDR20291_1632	putative hydrolase	-3.6675493	0.000231914	CD630_17350
CDR20291_1638A	hypothetical protein	-3.8583684	0.018924941	
CDR20291_1645A	conserved hypothetical protein	-4.4376742	0.005198032	CD630_17511
CDR20291_1647	ABC transporter, ATP-binding protein	-9.7070872	8.74E-07	CD630_17530
CDR20291_1648	putative ABC transporter, permease protein	-3.38361	0.000640915	CD630_17540
CDR20291_1649	putative ABC transporter, permease protein	-3.4847507	2.25E-06	CD630_17550
CDR20291_1662	glyceraldehyde-3-phosphate dehydrogenase 1	-12.425267	4.04E-18	CD630_17670
CDR20291_1669	amino acid ABC transporter, substrate-binding protein	-4.082594	0.01184514	CD630_17740
CDR20291_1671	amino acid ABC transporter, ATP-binding protein	-3.3703004	0.00035721	CD630_17760
CDR20291_1674	conserved hypothetical protein	-10.928471	5.32E-09	CD630_17790

CDR20291_1680	argininosuccinate synthase	-3.4458724	1.11E-22	CD630_17850
CDR20291_1683	putative membrane protein	-9.1299109	4.09E-05	CD630_17880
CDR20291_1684	putative membrane protein	-3.3319461	0.000531965	CD630_17890
CDR20291_1688	putative membrane protein	-10.718819	4.59E-09	CD630_17930
CDR20291_1690	conserved hypothetical protein	-10.932953	3.14E-09	CD630_17950
CDR20291_1701	putative fructokinase	-2.6070016	3.89E-08	CD630_18060
CDR20291_1707	putative two-component system response regulator (partial)	-3.4106667	0.001949804	CD630_18120
CDR20291_1710	conserved hypothetical protein	-4.2619905	8.18E-06	CD630_18150
CDR20291_1715	adenine deaminase	-2.3565943	2.49E-06	CD630_18200
CDR20291_1717	conserved hypothetical protein, UPF0246 family	-3.7986282	1.08E-54	CD630_18230
CDR20291_1729	3-phosphoshikimate 1-carboxyvinyltransferase	-4.1256249	2.03E-31	CD630_18340
CDR20291_1730	chorismate synthase (5-enolpyruvylshikimate-3-phosphate phospholyase)	-3.6545564	2.72E-12	CD630_18350
CDR20291_1731	P-protein [includes: chorismate mutase and prephenate dehydratase]	-2.3917393	1.05E-08	CD630_18360
CDR20291_1732	shikimate dehydrogenase	-3.5549037	1.96E-06	CD630_18370
CDR20291_1733	shikimate kinase	-3.3028453	0.000492854	CD630_18380
CDR20291_1740	putative rna methyltransferase	-10.617979	2.48E-08	CD630_18441
CDR20291_1742	putative conjugative transposon protein	-10.473184	5.32E-08	
CDR20291_1752	putative lantibiotic abc transporter, permease protein	-9.490417	2.12E-06	
CDR20291_1763	replicative dna helicase	-8.6325145	3.65E-05	
CDR20291_1776	putative conjugal transfer protein	-10.166418	2.40E-07	
CDR20291_1777	hypothetical protein	-9.7034527	4.44E-06	
CDR20291_1791	conserved hypothetical protein	-10.834978	2.00E-09	
CDR20291_1800	putative conjugative transposon mobilization protein	-5.7792859	9.72E-05	
CDR20291_1801	putative exported protein	-13.299252	1.29E-36	
CDR20291_1810	uncharacterised protein	-10.027069	1.51E-07	CD630_18800
CDR20291_1811	Fragment of ABC-type transport system,substrate-binding protein	-3.1963323	2.77E-05	CD630_18910
CDR20291_1813	putative regulatory protein	-6.4931452	1.86E-06	CD630_18930
CDR20291_1824	ABC transporter, permease protein	-11.062266	9.05E-09	CD630_19040
CDR20291_1834	putative ethanolamine/propanediol ammonia-lyase heavy chain	-2.2511782	0.02419512	CD630_19130
CDR20291_1836	putative ethanolamine/propanediol utilization protein	-8.7303983	2.68E-05	CD630_19150
CDR20291_1843	putative ethanolamine/propanediol utilization protein	-3.3499871	0.036263551	CD630_19220
CDR20291_1844	putative ethanolamine/propanediol utilization protein	-10.374654	3.87E-08	CD630_19230
CDR20291_1846	putative ethanolamine/propanediol utilization protein	-9.6101282	1.08E-06	CD630_19250
CDR20291_1850	putative aminotransferase	-2.2535472	3.03E-05	
CDR20291_1851	putative membrane protein	-5.1365417	6.87E-30	CD630_19280
CDR20291_1864	uncharacterised protein	-6.3161014	5.05E-07	CD630_19410
CDR20291_1866	uncharacterised protein	-2.0887477	0.020915882	CD630_19430
CDR20291_1868	putative glyoxalase	-3.3869105	3.09E-05	CD630_19450
CDR20291_1872	putative two-component sensor histidine kinase	-2.1801402	9.51E-06	CD630_19490

CDR20291_1878	ABC transporter, ATP-binding protein	-4.3799616	0.007351074	CD630_19550
CDR20291_1880	two-component response regulator	-2.0640702	0.026515321	CD630_19570
CDR20291_1888	putative membrane protein	-4.5371933	2.26E-08	CD630_19650
CDR20291_1889	putative acyl-CoA thioesterase	-12.347925	2.85E-16	CD630_19660
CDR20291_1891	putative membrane protein	-2.0885502	3.98E-13	CD630_19680
CDR20291_1894	putative protein, Sir2 family; putative silencing, chromosome stability or ageing; putative ADP ribosyltran	-2.2369375	4.38E-06	CD630_19710
CDR20291_1898	tRNA delta(2)-isopentenylpyrophosphate transferase	-6.9093233	1.70E-07	CD630_19750
CDR20291_1907	Fragment of putative phenazine biosynthesis protein	-2.4903042	0.003317094	CD630_19820
CDR20291_1910	putative radical sam protein	-2.0854042	5.11E-19	CD630_19860
CDR20291_1911	cell surface protein	-5.2165166	0.000445038	CD630_19870
CDR20291_1912	putative tryptophan transport protein	-5.2366328	0.000437028	CD630_19880
CDR20291_1913	putative membrane protein	-11.006895	1.51E-09	CD630_19890
CDR20291_1921	putative membrane protein	-3.0238729	1.22E-12	CD630_19950
CDR20291_1929	conserved hypothetical protein	-12.014734	1.61E-13	CD630_20160
CDR20291_1931	AraC-family transcriptional regulator	-2.6925282	6.25E-10	CD630_20180
CDR20291_1934	conserved hypothetical protein	-3.4789462	3.55E-16	CD630_20210
CDR20291_1943	ornithine carbamoyltransferase	-9.977246	4.16E-07	CD630_20300
CDR20291_1945	acetylglutamate kinase	-4.4900742	0.000366958	CD630_20320
CDR20291_1947	N-acetyl-gamma-glutamyl-phosphate reductase	-3.784434	0.02482962	CD630_20340
CDR20291_1949	putative N-acetyltransferase	-4.072094	3.00E-10	
CDR20291_1951	putative hydrolase	-3.8419226	0.021007837	CD630_20430
CDR20291_1953	putative transcriptional regulator	-2.1118201	0.013169261	CD630_20450
CDR20291_1955	RpiR-family transcriptional regulator	-4.1922098	0.003699725	CD630_20480
CDR20291_1975	ABC transporter, ATP-binding protein	-2.4553517	4.09E-17	CD630_20680
CDR20291_1988	xanthine dehydrogenase iron-sulfur binding subunit	-9.9366736	2.17E-07	CD630_20810
CDR20291_1993	putative iron-sulfur flavoprotein	-2.1366208	0.017496433	CD630_20860
CDR20291_2009A	conserved hypothetical protein	-7.6916051	0.000355235	CD630_21021
CDR20291_2011	putative ABC transporter, permease protein	-2.0940096	8.07E-11	CD630_21040
CDR20291_2019	uncharacterised protein	-7.7436136	9.05E-09	CD630_21120
CDR20291_2021	two-component system response regulator	-2.9716981	1.17E-09	CD630_21140
CDR20291_2022A	uncharacterised protein	-9.6880104	1.70E-06	CD630_21151
CDR20291_2024	thioredoxin reductase	-2.5181793	1.39E-06	CD630_21170
CDR20291_2027	putative 2-nitropropane dioxygenase	-5.5998864	6.38E-06	CD630_21200
CDR20291_2033	putative membrane protein	-2.7937195	2.46E-07	CD630_21260
CDR20291_2043	conserved hypothetical protein	-8.6481252	4.75E-05	CD630_21361
CDR20291_2048	D-alanyl-D-alanine carboxypeptidase	-4.9225481	0.001467824	CD630_21410
CDR20291_2056A	conserved hypothetical protein	-2.8935145	0.000429551	CD630_21501
CDR20291_2080	probable amino-acid ABC transporter, substrate-binding protein	-2.1985778	3.92E-09	CD630_21740
CDR20291_2084	putative iron- sulfur subunit of hydrogenase	-3.8280441	1.01E-12	CD630_21780

CDR20291_2088	ATP-dependent RNA helicase	-3.7428112	3.96E-09	CD630_21830
CDR20291_2090	conserved hypothetical protein	-4.3503167	1.18E-06	CD630_21850
CDR20291_2100	putative regulator of a specific sugar metabolism	-2.8239443	4.05E-18	CD630_21940
CDR20291_2104	putative subunit of oxidoreductase	-3.8487376	0.013370592	CD630_21980
CDR20291_2105	putative subunit of oxidoreductase	-4.662126	8.83E-06	CD630_21990
CDR20291_2107	putative histidinol-phosphate aminotransferase	-2.0576684	0.036443135	CD630_22000
CDR20291_2108	lactose permease	-9.2484645	6.23E-11	CD630_22010
CDR20291_2111	probable esterase	-3.6063187	0.013224119	CD630_22040
CDR20291_2121	putative regulatory protein	-12.670197	6.92E-21	CD630_22140
CDR20291_2124	3-dehydroquinate dehydratase	-11.084701	6.97E-10	CD630_22170
CDR20291_2130	anaerobic sulfite reductase subunit C	-2.1313927	0.000425926	CD630_22310
CDR20291_2131	anaerobic sulfite reductase subunit B	-12.377263	3.95E-17	CD630_22320
CDR20291_2136	conserved hypothetical protein	-2.3867538	0.000569291	CD630_22370
CDR20291_2138	putative sodium:solute symporter	-5.9709701	3.82E-07	CD630_22390
CDR20291_2139	N-acetylneuraminate lyase	-11.619583	3.30E-11	CD630_22400
CDR20291_2149	putative ATPase	-2.4578884	0.02173394	CD630_22490
CDR20291_2154	putative amino acid recemase	-3.9650669	0.013425787	CD630_22540
CDR20291_2156	putative PTS system, EIIc component	-11.778832	2.25E-12	CD630_22560
CDR20291_2167	PTS system, IIbc component	-2.7669111	0.003439136	CD630_22690
CDR20291_2175	sugar phosphate aldolase	-11.534209	2.45E-11	CD630_22770
CDR20291_2176	L-ribulose-5-phosphate 4-epimerase	-3.327779	0.040792155	CD630_22780
CDR20291_2178	PTS system, IIc component	-2.6455975	8.11E-06	CD630_22800
CDR20291_2179	PTS system, IIb component	-10.670977	6.02E-09	CD630_22810
CDR20291_2182	conserved hypothetical protein	-3.1817851	6.83E-12	CD630_22840
CDR20291_2186	putative membrane protein	-11.630066	8.31E-12	CD630_22870
CDR20291_2213	putative galactitol-1-phosphate 5-dehydrogenase	-11.062213	3.51E-09	CD630_23240
CDR20291_2220	mannitol-1-phosphate 5-dehydrogenase	-4.403616	3.56E-18	CD630_23310
CDR20291_2223	PTS system, mannitol-specific IIbc component	-2.3862024	0.000727899	CD630_23340
CDR20291_2228	4-hydroxybutyrate CoA transferase	-3.139444	1.35E-22	CD630_23390
CDR20291_2234	LysR-family regulatory protein	-2.9641958	4.86E-07	CD630_23450
CDR20291_2236	putative Xaa-Pro dipeptidase	-2.7409617	0.000268393	CD630_23470
CDR20291_2237	glycine/sarcosine/betaine reductase complex component C alpha subunit	-10.348809	2.24E-07	CD630_23480
CDR20291_2238	glycine/sarcosine/betaine reductase complex component C beta subunit	-12.040825	5.76E-14	CD630_23490
CDR20291_2238A	hypothetical protein	-8.7855494	2.94E-05	
CDR20291_2239	glycine reductase complex component B gamma subunit	-5.7136895	6.26E-05	CD630_23510
CDR20291_2240	glycine/sarcosine/betaine reductase complex component A	-11.409042	2.67E-11	CD630_23520
CDR20291_2241	glycine reductase complex component B alpha and beta subunits	-7.6874818	7.70E-26	CD630_23540
CDR20291_2242	thioredoxin	-8.2261778	0.000102464	CD630_23550
CDR20291_2243	thioredoxin reductase	-12.145871	1.16E-14	CD630_23560

CDR20291_2249	abc transporter, permease protein	-2.5266721	4.90E-08	CD630_23620
CDR20291_2258	L-aspartate oxidase	-2.8908191	4.80E-05	CD630_23710
CDR20291_2259	quinolinate synthetase A	-12.589537	9.22E-21	CD630_23720
CDR20291_2262	uncharacterised protein	-2.1407735	0.006204169	CD630_23750
CDR20291_2267	putative indolepyruvate oxidoreductase subunit	-3.512499	0.031863345	CD630_23800
CDR20291_2281	putative membrane protein	-3.2000688	3.33E-13	CD630_23910
CDR20291_2282	putative tRNA-binding protein	-9.6713704	6.86E-12	CD630_23920
CDR20291_2289	putative spore coat protein	-8.1535208	0.000118375	CD630_23990
CDR20291_2299	transposase	-9.0979844	9.32E-06	
CDR20291_2301	pyruvate, phosphate dikinase	-15.391292	7.44E-124	CD630_24100
CDR20291_2302	putative pyruvate, phosphate dikinase regulatory protein	-9.7044456	4.98E-12	CD630_24110
CDR20291_2303	putative transcriptional repressor ccpn	-4.6579716	6.64E-20	CD630_24120
CDR20291_2305	PTS system, IIa component	-9.9448657	1.11E-06	CD630_24140
CDR20291_2306	putative sorbitol-like operon activator protein (Glucitol)	-2.9069618	0.004163173	CD630_24150
CDR20291_2308	PTS system, glucitol/sorbitol-specific IIbc component	-2.3629065	0.000153578	CD630_24170
CDR20291_2310	AraC-family transcriptional regulator	-2.2129957	3.16E-05	CD630_24190
CDR20291_2311	conserved hypothetical protein	-2.2923252	1.51E-07	CD630_24200
CDR20291_2316	phosphate butyryltransferase	-5.2606181	0.000524157	CD630_24250
CDR20291_2327	DNA repair protein	-10.240062	5.68E-07	CD630_24350
CDR20291_2334	putative stage IV sporulation protein	-3.6171875	1.40E-49	CD630_24420
CDR20291_2335	putative sporulation protein yqfc	-9.0043923	5.47E-05	CD630_24430
CDR20291_2338	putative tRNA binding protein	-5.9504504	4.18E-05	CD630_24460
CDR20291_2341	putative radical SAM superfamily protein	-7.8541487	4.03E-23	CD630_24480
CDR20291_2342	putative methyltransferase	-5.864619	1.13E-23	CD630_24490
CDR20291_2345	putative CRISPR-associated protein	-3.867968	1.72E-08	CD630_24520
CDR20291_2346	putative CRISPR-associated negative autoregulator	-6.752823	9.41E-09	CD630_24530
CDR20291_2353	chaperone protein	-4.5047141	0.005621238	CD630_24600
CDR20291_2356	heat-inducible transcription repressor	-7.2351693	2.18E-16	CD630_24630
CDR20291_2361	putative exported protein	-3.4753529	0.000623817	CD630_24680
CDR20291_2362	putative stage II sporulation protein P	-11.893307	5.43E-12	CD630_24690
CDR20291_2363	germination protease	-10.866403	2.00E-09	CD630_24700
CDR20291_2376	putative exported protein	-10.705959	7.97E-09	CD630_24830
CDR20291_2384	mannose-6-phosphate isomerase	-8.4224361	0.000120381	CD630_24910
CDR20291_2386	selenocysteine-specific elongation factor	-11.962276	1.62E-75	CD630_24930
CDR20291_2387	L-seryl-tRNA(Sec) selenium transferase (selenocysteinyl-tRNA(Sec) synthase)	-10.999944	2.46E-32	CD630_24950
CDR20291_2388	selenide,water dikinase	-12.739132	6.13E-24	CD630_24960
CDR20291_2389	putative competence protein	-11.202403	2.82E-10	CD630_24970
CDR20291_2403	transcriptional antiterminator	-5.3143299	0.000436624	CD630_25110
CDR20291_2404	PTS system, IIa component	-6.8157987	4.30E-06	CD630_25120

CDR20291_2405	putative translation inhibitor endoribonuclease	-9.9417142	1.99E-06	CD630_25130
CDR20291_2412	putative metal dependent phosphohydrolase	-11.297812	2.34E-09	CD630_25230
CDR20291_2418	putative membrane protein	-4.0299175	0.016987686	CD630_25310
CDR20291_2426	ribonuclease Z	-10.8226	2.61E-09	CD630_25390
CDR20291_2427	NADH oxidase	-3.7040065	1.41E-42	CD630_25400
CDR20291_2428	sodium:dicarboxylate symporter family protein	-3.0517887	2.53E-20	CD630_25410
CDR20291_2438	putative phosphosugar isomerase/binding protein	-2.8180186	6.38E-05	CD630_25520
CDR20291_2439	phosphotransferase system, C component	-4.7117784	2.49E-06	CD630_25530
CDR20291_2440	phosphotransferase system, B component	-9.3272539	3.52E-06	CD630_25550
CDR20291_2444	rna methyltransferase, rsmd family	-8.2807671	0.000170066	CD630_25590
CDR20291_2446	putative phosphatase	-2.0509633	9.93E-14	CD630_25610
CDR20291_2450	MerR-family transcriptional regulator	-2.3581326	0.000202293	CD630_25640
CDR20291_2452	PTS system, IIa component	-2.7616728	0.013722841	CD630_25660
CDR20291_2456	two-component sensor histidine kinase	-4.3090347	2.65E-14	CD630_25700
CDR20291_2467	ribosomal RNA small subunit methyltransferase	-4.2066201	8.63E-41	CD630_25810
CDR20291_2469	putative membrane protein	-2.1974146	3.06E-13	CD630_25830
CDR20291_2479	fibronectin-binding protein	-3.6911719	3.55E-08	CD630_25920
CDR20291_2482	PyrR bifunctional protein	-3.6320033	9.72E-05	CD630_25950
CDR20291_2483	putative pseudouridylate synthase	-5.2947999	4.99E-32	CD630_25960
CDR20291_2484	lipoprotein signal peptidase	-3.1852481	9.27E-10	CD630_25970
CDR20291_2494	5'-methylthioadenosine/S- adenosylhomocysteine nucleosidase	-3.1920874	0.018485224	CD630_26110
CDR20291_2508	putative exported protein	-3.8762875	1.60E-10	CD630_26240
CDR20291_2513	stage IV sporulation protein A	-10.117047	2.19E-64	CD630_26290
CDR20291_2516	cobalt dependent x-pro dipeptidase	-3.429234	6.17E-19	
CDR20291_2530	RNA polymerase sigma-G factor	-11.244002	2.32E-10	CD630_26420
CDR20291_2531	RNA polymerase sigma-E factor	-11.156865	5.97E-10	CD630_26430
CDR20291_2532	sporulation sigma-E factor processing peptidase	-12.622688	8.35E-20	CD630_26440
CDR20291_2535	putative membrane protein	-3.6924701	1.14E-16	CD630_26470
CDR20291_2538	putative cell division protein	-2.7030835	3.95E-08	CD630_26500
CDR20291_2540	cell division/stage V sporulation protein	-11.963422	3.28E-13	CD630_26520
CDR20291_2544	stage V sporulation protein D (sporulation specific penicillin-binding protein)	-9.7570052	1.34E-93	CD630_26560
CDR20291_2545	putative membrane protein	-11.204023	2.58E-10	CD630_26570
CDR20291_2547	prolipoprotein diacylglyceryl transferase	-4.019416	8.64E-09	CD630_26590
CDR20291_2549	probable peptidase	-3.6457999	7.54E-25	CD630_26610
CDR20291_2550	putative GTP-binding protein	-2.4687371	0.00090809	CD630_26620
CDR20291_2555	PTS system, glucose-specific IIbc component	-11.141219	3.16E-09	CD630_26670
CDR20291_2556	putative transcription antiterminator	-3.3218843	0.00133179	CD630_26680
CDR20291_2558	oligopeptide ABC transporter, ATP-binding protein	-5.169571	3.16E-15	CD630_26700
CDR20291_2559	oligopeptide transporter, ATP-binding protein	-4.0626759	5.90E-05	CD630_26710

CDR20291_2569	putative Calcium-chelating exported protein	-8.9340908	1.48E-05	CD630_26810
CDR20291_2570	pyruvate-flavodoxin oxidoreductase	-5.1476397	0.000118426	CD630_26820
CDR20291_2573	putative regulatory protein	-9.2798079	2.64E-86	CD630_26850
CDR20291_2574	putative membrane protein	-4.5967899	8.56E-06	CD630_26860
CDR20291_2575	conserved hypothetical protein	-2.5918402	0.001545382	CD630_26870
CDR20291_2584	putative aminotransferase	-3.1166896	0.001183274	CD630_26960
CDR20291_2585	putative amidohydrolase	-6.7686179	4.34E-08	CD630_26970
CDR20291_2586	putative permease	-5.6950739	0.000142886	CD630_26980
CDR20291_2587	putative membrane protein	-4.2325168	8.10E-05	CD630_26990
CDR20291_2610	two-component sensor histidine kinase	-6.6711311	5.72E-83	CD630_27210
CDR20291_2611	two-component response regulator	-12.477401	2.39E-97	CD630_27220
CDR20291_2613	probable polysaccharide deacetylase	-11.699848	6.90E-64	CD630_27240
CDR20291_2614	putative UDP-NAM-NAG-(pentapeptide) pyrophosphoryl-undecaprenol N-acetylglucosamine transferase	-3.4373396	1.41E-42	CD630_27250
CDR20291_2618	putative regulator of the sigma(E) factor	-11.347095	1.40E-10	CD630_27290
CDR20291_2619	conserved hypothetical protein	-6.0667062	1.93E-05	CD630_27300
CDR20291_2620	putative membrane protein	-3.9902957	8.37E-09	CD630_27310
CDR20291_2634	adenine phosphoribosyltransferase	-10.405209	3.29E-08	CD630_27450
CDR20291_2638	putative radical SAM family protein	-11.421514	2.16E-11	CD630_27490
CDR20291_2640	accessory gene regulator	-2.1481229	1.52E-12	CD630_27500
CDR20291_2642	conserved hypothetical protein	-4.8800948	0.001850864	CD630_27520
CDR20291_2643	phosphoenolpyruvate-protein phosphotransferase	-12.58436	4.12E-20	CD630_27550
CDR20291_2644	PTS system, phosphocarrier protein	-8.5080537	8.07E-05	CD630_27560
CDR20291_2646	putative DNA modification methylase	-2.6739078	5.23E-27	CD630_27580
CDR20291_2654	putative penicillin-binding protein repressor	-9.935973	3.09E-07	CD630_27660
CDR20291_2667	putative mannose-1-phosphate guanylyltransferase	-8.6127756	3.37E-05	CD630_27790
CDR20291_2689	putative membrane protein	-4.5624533	0.004902293	CD630_28000
CDR20291_2691	queuine tRNA-ribosyltransferase	-2.1513639	0.00052262	CD630_28020
CDR20291_2693	S-adenosylmethionine:tRNA ribosyltransferase-isomerase	-2.6981693	3.21E-05	CD630_28040
CDR20291_2696	crossover junction endodeoxyribonuclease RuvC	-8.4361974	0.000103484	CD630_28070
CDR20291_2702	2-hydroxy-3-oxopropionate reductase	-9.7956307	4.45E-07	CD630_28130
CDR20291_2709	transposase	-3.2792734	0.042275738	
CDR20291_2711	putative membrane protein	-11.066016	9.28E-10	CD630_28210
CDR20291_2712	putative peptidase	-7.0920009	4.24E-12	CD630_28220
CDR20291_2714	putative membrane protein	-3.3436557	3.06E-05	
CDR20291_2729	putative two-component response regulator	-2.7986726	6.08E-05	CD630_28380
CDR20291_2736	rubrerythrin	-2.3888036	2.25E-08	CD630_28450
CDR20291_2751	putative membrane protein	-12.719602	3.27E-23	CD630_28600
CDR20291_2752	putative membrane protein	-10.956661	5.95E-09	CD630_28610
CDR20291_2753	putative dipeptidase	-4.8143465	0.000955596	CD630_28620

CDR20291_2758	putative lantibiotic ABC transporter, ATP-binding protein	-9.1798129	1.46E-05	CD630_03640
CDR20291_2760	two-component system, sensor histidine kinase	-2.1773023	8.36E-05	CD630_03610
CDR20291_2770	putative drug/sodium antiporter	-2.6145223	8.19E-05	CD630_28740
CDR20291_2771	putative ferrichrome ABC transporter, ATP-binding protein	-11.077312	8.23E-10	CD630_28750
CDR20291_2776	PTS system, lichenan-specific IIa component	-6.8812841	5.14E-05	CD630_28800
CDR20291_2790	V-type sodium ATP synthase subunit G	-4.9408888	0.002261274	CD630_29561
CDR20291_2791	V-type sodium ATP synthase subunit C	-4.5449587	0.004016583	CD630_29570
CDR20291_2796	conserved hypothetical protein	-10.141353	1.76E-07	CD630_29620
CDR20291_2800	aldehyde-alcohol dehydrogenase	-3.2532502	1.54E-09	CD630_29660
CDR20291_2810	putative CRISPR-associated Cas2 family protein	-2.2808838	0.017481093	CD630_29750
CDR20291_2811	putative CRISPR-associated Cas1 family protein	-2.0996766	7.03E-16	CD630_29760
CDR20291_2812	putative CRISPR-associated Cas4 family protein	-3.8525046	7.52E-09	CD630_29770
CDR20291_2814	putative CRISPR-associated Cas5 family protein	-3.9032115	2.08E-28	CD630_29790
CDR20291_2816	putative CRISPR-associated protein, CXXC-CXXC	-3.4462443	6.98E-21	CD630_29810
CDR20291_2817	putative CRISPR-associated Cas6 family protein	-4.6964108	7.96E-08	CD630_29820
CDR20291_2820	ABC transporter, ATP-binding protein	-2.3666076	0.007825733	CD630_29850
CDR20291_2821	undecaprenyl-diphosphatase	-2.9827166	0.005321511	CD630_29860
CDR20291_2823	two-component system esponse regulator	-12.425425	6.02E-18	CD630_29880
CDR20291_2826	putative ABC transporter, permease protein	-4.1852387	3.91E-12	CD630_29910
CDR20291_2827	conserved hypothetical protein	-2.061318	3.85E-05	CD630_29920
CDR20291_2836	putative dihydrodipicolinate synthase	-4.9681551	1.21E-16	CD630_30000
CDR20291_2838	probable carbohydrate hydrolase	-4.3720453	1.32E-17	CD630_30020
CDR20291_2839	probable carbohydrate hydrolase (N-terminus)	-11.768738	2.29E-12	CD630_30030
CDR20291_2840	putative carbohydrate permease	-11.516867	8.87E-12	CD630_30040
CDR20291_2844	Fragment of conserved hypothetical protein (N-terminal region)	-3.8663419	5.53E-12	CD630_30080
CDR20291_2848	putative glycosyl hydrolase	-3.2928482	2.28E-31	CD630_30120
CDR20291_2856	Fragment of conserved hypothetical protein,DUF1848 family	-4.1962539	6.77E-27	CD630_30200
CDR20291_2862	PTS system, glucose-specific IIa component	-4.3233516	2.27E-06	CD630_30270
CDR20291_2863	putative phosphosugar isomerase	-5.1849616	0.000627812	CD630_30280
CDR20291_2865	PTS system, maltose and glucose-specific IIbc component	-3.5140984	1.41E-08	CD630_30300
CDR20291_2868	thioredoxin	-10.970905	3.11E-08	CD630_30330
CDR20291_2874	putative ATPase	-3.7948625	7.90E-06	CD630_30390
CDR20291_2877	putative membrane protein	-2.2545385	5.47E-16	CD630_30420
CDR20291_2883	PTS system, IIc component	-11.342006	6.86E-11	CD630_30480
CDR20291_2885	putative membrane protein	-2.1044696	7.14E-05	CD630_30500
CDR20291_2890	ABC transporter, ATP-binding protein	-4.7596734	6.25E-10	CD630_30560
CDR20291_2895	PTS system, IIbc component	-11.244827	2.58E-10	CD630_30610
CDR20291_2898	xylose isomerase	-2.2453444	7.28E-05	CD630_30640
CDR20291_2910A	hypothetical protein	-2.1936052	7.62E-11	

CDR20291_2916	phosphosugar-binding transcriptional regulator	-2.0706985	0.004525013	CD630_30770
CDR20291_2918	6-phospho-beta-glucosidase	-5.1835801	0.000232599	CD630_30790
CDR20291_2922	transcription antiterminator	-2.2293826	4.48E-07	CD630_30830
CDR20291_2930	trehalose-6-phosphate hydrolase	-10.917541	7.51E-09	CD630_30910
CDR20291_2935	PTS system, IIabc component	-2.406143	1.91E-05	CD630_30970
CDR20291_2939	PTS system, IIbc component	-3.4264691	0.000722244	CD630_31010
CDR20291_2957	putative exported protein	-3.0368921	2.48E-07	CD630_31180
CDR20291_2962	predicted enoate reductase	-4.0993423	3.65E-15	
CDR20291_2964	transcriptional regulator, padr-like family	-2.3288528	0.001252172	
CDR20291_2965	putative flavodoxin	-2.2116619	6.64E-07	CD630_31210
CDR20291_2966	ArsR-family transcriptional regulator	-5.7622049	5.05E-05	CD630_31220
CDR20291_2967	putative nitroreductase	-4.2444797	1.07E-24	CD630_31230
CDR20291_2976	PTS system, IIa component	-2.9001677	5.67E-05	CD630_31320
CDR20291_2978	PTS system, IIabc component	-2.283737	0.000276631	CD630_31340
CDR20291_2983A	Transcriptional regulator, Phage-type	-8.3469313	1.32E-06	CD630_31390
CDR20291_2983B	putative membrane protein	-2.1047877	3.56E-13	CD630_31400
CDR20291_2985	putative transcriptional regulator	-2.5602737	4.72E-11	CD630_31440
CDR20291_2988	hypothetical protein	-3.3646465	1.06E-13	
CDR20291_2993	hypothetical protein	-9.842435	7.79E-07	
CDR20291_2994	crispr-associated helicase cas3	-2.3609204	7.63E-21	
CDR20291_2995	crispr-associated autoregulator, devr family	-3.5524792	3.39E-14	
CDR20291_2999	hypothetical protein	-3.7948936	6.39E-06	
CDR20291_2999A	hypothetical protein	-10.066082	1.32E-07	
CDR20291_3001	putative phage protein	-12.140992	1.38E-14	CD630_31510
CDR20291_3002	conserved hypothetical protein	-10.998107	1.60E-09	CD630_31520
CDR20291_3003	putative phage transcriptional regulator	-9.2487412	7.27E-06	CD630_31521
CDR20291_3004	putative phage DNA-binding protein	-9.2969411	4.27E-06	CD630_31530
CDR20291_3013	putative acetyltransferase	-2.5610565	4.01E-06	CD630_31590
CDR20291_3017	conserved hypothetical protein	-2.183812	0.015448132	CD630_31630
CDR20291_3019	uncharacterised protein	-9.8547377	1.18E-06	CD630_31650
CDR20291_3020	putative transcriptional regulator	-2.0360951	0.040671178	CD630_31660
CDR20291_3022	conserved hypothetical protein	-2.8332074	0.00605395	CD630_31680
CDR20291_3023	putative transporter	-10.441675	4.48E-20	CD630_31690
CDR20291_3031	central glycolytic genes regulator	-10.319368	3.91E-07	CD630_31750
CDR20291_3032	RNA polymerase sigma-54 factor	-10.87862	1.91E-09	CD630_31760
CDR20291_3034	D-hydantoinase	-2.6819636	1.05E-09	CD630_31780
CDR20291_3035	putative dihydroorotate dehydrogenase, catalytic subunit	-2.0669713	0.007351074	CD630_31790
CDR20291_3036	xanthine permease	-2.551315	0.000603556	CD630_31800
CDR20291_3039	putative amidohydrolase	-4.2564068	1.95E-27	CD630_31830

CDR20291_3045	putative iron-sulfur flavoprotein	-2.329184	0.00139838	CD630_31890
CDR20291_3046	MerR-family transcriptional regulator	-4.6323914	6.03E-05	CD630_31900
CDR20291_3051	possible sensor histidine kinase	-7.1067582	8.48E-11	
CDR20291_3056	putative penicillin-binding protein	-2.6677763	0.000881026	CD630_31960
CDR20291_3059	putative ABC transporter, permease protein	-3.0474555	1.21E-05	CD630_31990
CDR20291_3061	ABC transporter, ATP-binding protein	-6.0897362	9.42E-06	CD630_32010
CDR20291_3069	conserved hypothetical protein	-2.3557632	0.001696595	CD630_32100
CDR20291_3074	putative osmoprotectant acid ABC transporter, ATP-binding protein	-3.2809222	0.000981009	CD630_32150
CDR20291_3078	chaperonin	-5.0826868	1.04E-15	CD630_32190
CDR20291_3079	putative methyltransferase	-4.3757583	0.002262804	CD630_32200
CDR20291_3083	dihydrodipicolinate synthase	-3.2898646	4.83E-21	CD630_32230
CDR20291_3092	conserved hypothetical protein, UPF0597 family	-3.0694132	4.37E-05	CD630_32320
CDR20291_3095	single-stranded DNA binding protein	-4.215445	4.42E-06	CD630_32350
CDR20291_3097	putative proline racemase	-6.2922358	5.28E-06	CD630_32370
CDR20291_3101	proline reductase	-10.853749	2.18E-09	CD630_32410
CDR20291_3102	conserved hypothetical protein	-10.042319	3.64E-07	CD630_32430
CDR20291_3103	proline reductase subunit proprotein	-11.96308	3.50E-13	CD630_32440
CDR20291_3104	sigma-54-dependent transcriptional activator	-6.8726207	4.05E-30	CD630_32450
CDR20291_3105	putative electron transfer protein	-5.2982155	0.000161875	CD630_32470
CDR20291_3107	small acid-soluble spore protein B	-6.9905882	4.11E-05	CD630_32490
CDR20291_3113	two-component response regulator	-2.917973	1.41E-33	CD630_32550
CDR20291_3115	putative polysaccharide deacetylase	-3.1469685	1.79E-13	CD630_32570
CDR20291_3116	iron-only hydrogenase	-2.9576056	2.76E-05	CD630_32580
CDR20291_3117	putative membrane protein	-2.0329158	2.49E-15	CD630_32590
CDR20291_3118	putative phosphate transport system protein	-2.6445892	0.000155749	CD630_32600
CDR20291_3132	conserved hypothetical protein	-9.50049	9.67E-06	CD630_32710
CDR20291_3133	putative membrane protein	-4.2705216	0.010534144	CD630_32720
CDR20291_3134	putative ferrous iron transport protein A	-3.9029582	4.61E-08	CD630_32730
CDR20291_3135	putative ferrous iron transport protein B	-3.0324582	5.63E-27	CD630_32740
CDR20291_3138	PTS system, IIc component	-10.928494	1.52E-09	CD630_32770
CDR20291_3140	PTS system, IIb component	-5.1459732	0.000807269	CD630_32790
CDR20291_3152	putative exported protein	-6.3497747	1.48E-07	CD630_32910
CDR20291_3153	putative membrane protein	-3.5117255	0.000108866	CD630_32920
CDR20291_3154	putative pilus assembly protein	-3.9386913	2.43E-07	CD630_32930
CDR20291_3155	putative type IV pilin	-4.546177	7.40E-06	CD630_32940
CDR20291_3156	putative type IV pilus-assembly protein	-3.3394235	6.53E-10	CD630_32950
CDR20291_3157	putative type IV pilus-assembly protein	-2.3135129	1.04E-11	CD630_32960
CDR20291_3159	putative ATP/GTP-binding protein	-4.4567502	0.002833391	CD630_32980
CDR20291_3160	probable transporter	-7.1734412	1.81E-21	CD630_32990

CDR20291_3163	chromate transport protein	-11.545148	4.56E-10	CD630_33020
CDR20291_3164	chromate transport protein	-11.416194	2.31E-11	CD630_33030
CDR20291_3167	trigger factor	-2.629523	0.010606117	CD630_33060
CDR20291_3169	ribonuclease Ph	-3.1011272	3.21E-05	CD630_33080
CDR20291_3172	conserved hypothetical protein	-2.6463275	1.74E-12	CD630_33110
CDR20291_3174	electron transport protein	-2.0019696	3.19E-10	CD630_33130
CDR20291_3177	conserved hypothetical protein	-3.5677684	9.14E-11	CD630_33151
CDR20291_3181	two-component response regulator	-2.639447	4.70E-10	CD630_33200
CDR20291_3183	conserved hypothetical protein	-2.1847543	3.72E-06	CD630_33220
CDR20291_3185	abc transporter, atp-binding protein	-3.0224801	2.99E-12	
CDR20291_3187	accessory gene regulator	-5.4458902	8.50E-20	
CDR20291_3197	GntR-family transcriptional regulator	-3.0486437	4.38E-19	CD630_33530
CDR20291_3205	putative multidrug efflux protein	-2.2351303	1.89E-15	CD630_33610
CDR20291_3206	ABC transporter, permease protein	-2.448778	1.21E-20	CD630_33620
CDR20291_3208	ABC transporter, ATP-binding protein	-3.9055961	2.43E-24	CD630_33640
CDR20291_3212	putative GTPase	-3.2434978	7.81E-06	CD630_33680
CDR20291_3213A	conserved hypothetical protein	-2.4609883	0.000850949	CD630_33682
CDR20291_3214	conserved hypothetical protein	-2.6791876	5.85E-16	CD630_33690
CDR20291_3216	pyruvate kinase	-5.5790519	0.000153578	CD630_33940
CDR20291_3231	HPr(Ser) kinase/phosphorylase	-12.256858	1.99E-15	CD630_34090
CDR20291_3237	ABC transporter, permease protein	-2.2017776	0.03346615	CD630_34150
CDR20291_3257	cobyric acid synthase	-2.5120312	6.20E-11	CD630_34350
CDR20291_3266	PTS system, IIb component	-2.7192074	7.92E-12	CD630_34440
CDR20291_3267	PTS system, IIa component	-8.1903189	0.000108644	CD630_34450
CDR20291_3268	endoglucanase	-2.7913119	3.48E-13	CD630_34460
CDR20291_3271	putative tagatose-6-phosphate ketose/aldose isomerase	-3.4392175	3.12E-09	CD630_34490
CDR20291_3274	GntR-family transcriptional regulator	-3.5224106	3.90E-12	CD630_34520
CDR20291_3277	putative exported protein	-3.0833136	6.99E-05	CD630_02810
CDR20291_3278	putative exported protein	-12.274946	3.01E-15	
CDR20291_3280	hypothetical protein	-3.3737859	1.07E-09	
CDR20291_3282	hypothetical protein	-2.882911	0.000409339	
CDR20291_3283	hypothetical protein	-4.4388776	0.005927078	
CDR20291_3284	phage portal protein	-2.1563563	6.69E-07	
CDR20291_3284A	hypothetical protein	-2.3626531	0.012233237	
CDR20291_3286A	hypothetical protein	-2.4929718	3.28E-10	
CDR20291_3287	hypothetical protein	-4.6542654	0.002235476	
CDR20291_3293	putative exported protein	-8.891722	2.62E-05	CD630_34570
CDR20291_3295	transketolase, pyridine binding subunit	-2.9132522	0.001017629	CD630_34590
CDR20291_3296	transketolase, thiamine disphosphate-binding subunit	-4.6920328	0.002215223	CD630_34600

CDR20291_3321	protein methyltransferase	-11.227735	1.82E-10	CD630_34850
CDR20291_3327	stage II sporulation protein e	-8.5691356	5.28E-101	CD630_34900
CDR20291_3329	putative septum formation protein	-4.1651988	2.45E-08	CD630_34920
CDR20291_3331	putative spore protein	-3.8138802	0.000420947	CD630_34940
CDR20291_3336	stage V sporulation protein T	-6.9443228	9.31E-10	CD630_34990
CDR20291_3353	stage V sporulation protein G	-11.351414	4.38E-11	CD630_35160
CDR20291_3354	putative transcriptional repressor	-2.3802941	2.70E-15	CD630_35170
CDR20291_3356	putative exported protein	-11.960059	1.67E-74	CD630_35190
CDR20291_3364	putative iron ABC transporter, permease protein	-3.0355244	2.61E-17	CD630_35290
CDR20291_3365	putative iron ABC transporter, ATP-binding protein	-12.459034	1.57E-17	CD630_35300
CDR20291_3366	putative phophoesterase	-3.136741	4.18E-07	CD630_35310
CDR20291_3368	phosphonate ABC transporter, ATP-binding protein	-12.114972	4.80E-14	CD630_35330
CDR20291_3369	phosphonate ABC transporter, ATP-binding protein	-3.6816525	1.06E-10	CD630_35340
CDR20291_3371	putative phosphonate metabolism protein	-12.200722	5.95E-15	CD630_35360
CDR20291_3372	putative phosphonate metabolism protein	-2.5483268	0.000885527	CD630_35370
CDR20291_3373	putative phosphonate metabolism protein	-10.169386	1.41E-16	CD630_35380
CDR20291_3386	putative arginine decarboxylase	-10.597952	2.03E-24	CD630_35510
CDR20291_3388	conserved hypothetical protein	-12.084201	8.52E-14	CD630_35512
CDR20291_3396	cell division protein	-9.6294125	3.70E-32	CD630_35590
CDR20291_3400	putative spore cortex-lytic enzyme	-3.4945141	4.94E-05	CD630_35630
CDR20291_3401	stage II sporulation protein	-7.9350046	2.50E-29	CD630_35640
CDR20291_3402	GntR-family transcriptional regulator	-2.2598549	2.08E-15	CD630_35650
CDR20291_3404	putative cell wall hydrolase	-11.855732	9.86E-125	CD630_35670
CDR20291_3410	conserved hypothetical protein	-4.818207	6.17E-18	CD630_35720
CDR20291_3411	conserved hypothetical protein	-2.6239525	0.000712165	CD630_35730
CDR20291_3412	putative membrane protein	-13.084245	2.18E-32	CD630_35740
CDR20291_3413	putative sodium:solute symporter	-2.6263424	1.74E-14	CD630_35750
CDR20291_3414	probable transporter	-4.101492	6.16E-34	CD630_35760
CDR20291_3423	ABC transporter, ATP-binding protein	-3.4507011	0.000103179	CD630_35850
CDR20291_3424	two-component sensor histidine kinase	-2.1452664	0.004851489	CD630_35860
CDR20291_3432	conserved hypothetical protein	-4.8514038	4.07E-08	CD630_35940
CDR20291_3451	uncharacterised protein	-2.6893384	2.01E-07	CD630_36130
CDR20291_3454A	putative conjugative transposon protein DUF961 family Tn916-like, CTn1-Orf29	-8.7031207	2.67E-05	CD630_03840
CDR20291_3458	putative conjugative transposon FtsK_SpoIIIE-related protein	-8.2376322	1.34E-07	CD630_33890
CDR20291_3459	putative conjugative transposon replication initiation factor	-7.2348404	1.86E-07	CD630_03810
CDR20291_3464	conjugative transposon protein	-10.441334	2.63E-08	CD630_03740
CDR20291_3471	putative abc transporter, permease protein	-11.016683	1.93E-08	
CDR20291_3473	putative cation efflux protein	-11.530523	8.09E-12	
CDR20291_3473C	putative conjugative transposon protein Tn916-like, CTn1-Orf14	-8.9341856	3.86E-05	CD630_03690

CDR20291_3475	integrase	-3.4483986	0.037922831	CD630_03550
CDR20291_3479	conserved hypothetical protein	-2.3605117	1.30E-11	CD630_36170
CDR20291_3481	putative acetyltransferase	-2.6064268	2.22E-16	CD630_36190
CDR20291_3485	putative ABC transporter, ATP-binding protein	-2.2401332	5.98E-05	CD630_36230
CDR20291_3491	putative PTS system, IIb component	-9.195605	6.03E-06	CD630_36300
CDR20291_3494	putative membrane protein	-5.5774219	0.000184998	CD630_36360
CDR20291_3495	putative phage-related protein	-6.4512863	4.46E-06	CD630_36361
CDR20291_3497	conserved hypothetical protein	-10.470481	2.60E-21	CD630_36380
CDR20291_3527	selenium metabolism protein yedf	-8.7756798	1.54E-08	CD630_36670
CDR20291_3528	putative transcriptional regulator	-4.0536022	1.99E-46	CD630_36680
CDR20291_3530	putative selenocysteine lyase	-8.2372888	5.54E-43	CD630_36700
CDR20291_3535	glucose inhibited division protein A	-10.595039	4.72E-07	CD630_36750
CDR20291_3536	putative tRNA modification GTPase	-10.052609	1.54E-07	CD630_36760
CDR20291_3537	SpoIIIJ-associated protein	-9.8101108	5.47E-07	CD630_36770
CDR20291_3538	putative sporulation membrane protein	-8.6838153	3.08E-05	CD630_36780
CDR20291_3539	conserved hypothetical protein	-9.6981636	1.51E-06	CD630_36781
CDR20291_3548	putative exported protein	-11.980904	3.27E-13	CD630_00070
CDR20291_3549	putative membrane protein	-5.8561275	8.42E-06	CD630_00080
CDR20291_3550	anti-sigma-B factor antagonist	-10.048784	9.88E-15	CD630_00090
CDR20291_3551	anti-sigma-B factor (serine-protein kinase)	-3.5362854	5.25E-17	CD630_00100
CDR20291_3552	RNA polymerase sigma-B factor	-12.563834	4.83E-20	CD630_00110
