# Supplementary material for: High-Throughput Analysis of Gene Essentiality and Sporulation in Clostridium difficile
Source: mBio. 2015 Feb 24;6(2):e02383-14. doi: 10.1128/mBio.02383-14 (PMC4358009; doi:10.1128/mBio.02383-14)
Supplement: Table S3 — Sequencing data accession numbers [file mbo001152196st3.docx]

**TABLE S3** Accession numbers of raw fastq files

| **Sample type** | **Sample** | **Run accession** |
| --- | --- | --- |
| **630Δ*erm* library:** |  |  |
| Input library | Cdiff_630_Lib | ERR237766 |
| Pre-sporulation | Cdiff_630_2 | ERR245853 |
| Sporulated | Cdiff_630_3 | ERR245854 |
| Germinated | Cdiff_630_4 | ERR245855 |
|  |  |  |
| **R20291 library:** |  |  |
| Input library | Sample_1_1 | ERR377408 |
|  |  | ERR377416 |
|  | Sample_1_2 | ERR377409 |
|  |  | ERR377417 |
| Pre-sporulation | Sample_2_1 | ERR377410 |
|  |  | ERR377418 |
|  | Sample_2_2 | ERR377411 |
|  |  | ERR377419 |
| Sporulated | Sample_3_1 | ERR377412 |
|  |  | ERR377420 |
|  | Sample_3_2 | ERR377413 |
|  |  | ERR377421 |
